# Estimation of SARS-CoV-2 Viral Dynamics Parameters in Favipiravir-Treated Cynomolgus Macaques: Comparison of Individual and Group Average Model Fitting

**DOI:** 10.3390/pathogens15101007

**Published:** 2026-09-23

**Authors:** Aarush Shetty, Hana M. Dobrovolny

**Affiliations:** Department of Physics & Astronomy, Texas Christian University, Fort Worth, TX 76129, USA

**Keywords:** mathematical model, parameter estimation, identifiability, favipiravir, SARS-CoV-2

## Abstract

Favipiravir, an antiviral that inhibits viral RNA-dependent RNA polymerase, has demonstrated promise as a therapeutic for RNA viral infections such as SARS-CoV-2. Mathematical modeling of viral kinetics provides a tool for analyzing the progression of viral infections and the action of antiviral drugs. In the present investigation, the viral kinetics of SARS-CoV-2 infection in cynomolgus macaques treated with the antiviral drug favipiravir were analyzed using a target-cell-limited mathematical model of viral infection. The model parameters were estimated by fitting the model independently to each of the 30 individual animals across four treatment groups (untreated control, 100 mg/kg, 150 mg/kg, and 180 mg/kg). Bootstrap resampling was applied to quantify the parameter uncertainty, and Mann–Whitney U tests were used to compare the group-level parameter estimates between the control and each treated group. Despite broad trends in several parameters, no statistically significant differences were detected between the control and any favipiravir-dose group. We also fit the model to the group-averaged viral titer curves, which led to statistically significant differences in some of the parameters. These results highlight the danger in using averaged viral titers when there is substantial inter-animal variability in individual viral kinetics data, and they suggest that larger cohorts may be required to detect drug effects with statistical confidence at the individual animal level.

## 1. Introduction

Severe acute respiratory syndrome coronavirus 2 (SARS-CoV-2), the etiologic agent of COVID-19 [1,2], remains a global health burden despite widespread vaccination campaigns. Since the first cases appeared in late 2019, SARS-CoV-2 has infected hundreds of millions of individuals worldwide, causing substantial morbidity and mortality [3]. In addition, the emergence of immune-escape variants [4,5] is contributing to ongoing transmission even in vaccinated individuals [6], and many people develop symptoms that persist after acute infection [7,8], a phenomenon called long COVID, thus increasing the pressures on healthcare services [9]. Although vaccines have substantially reduced the disease severity and mortality [10,11], breakthrough infections remain common, especially in immunocompromised people, the elderly, and individuals with comorbid conditions [12].

The existing currently approved antiviral agents against SARS-CoV-2 infection are quite limited in both number and efficacy [13]. Remdesivir, which is one of the earlier available therapies [14,15], acts as an inhibitor of the virus’ RNA polymerase enzyme [16], but needs to be injected intravenously. As such, it is often administered to patients in hospitals and consequently is not used often for treatment in COVID-19’s early stages. While studies have shown some effectiveness in shortening hospital stays and reducing mortality, the effect is often most significant when remdesivir is administered early [17,18,19,20]. Paxlovid (nirmatrelvir/ritonavir) is an oral protease inhibitor [21] and was found to be more effective than remdesivir [22], but has the drawbacks of potential severe drug interactions [23] and viral rebound [24,25]. Another commonly used antiviral, molnupiravir, is an oral nucleoside analog [26,27] that achieved about a 30% decrease in the rate of hospitalizations [28], but it can also result in viral rebound [29,30]. Monoclonal antibodies, which used to be regarded as one of the pillars of COVID-19 therapy [31], have lost some of their potency due to viral evolution [32], although they still provide some protection from severe illness [33,34]. While there are still viable antiviral treatment options for SARS-CoV-2, the drawbacks of a narrow therapeutic window, delivery mode restrictions, pharmacodynamic and pharmacokinetic interactions [35], and the possible emergence of antiviral resistance [36] are strong indicators that other antivirals should be developed.

Favipiravir is an orally available broad-spectrum antiviral nucleoside analog, initially designed to treat influenza [37], that has since been investigated as a therapeutic agent for SARS-CoV-2 [38,39]. Following its phosphorylation to its active form, favipiravir inhibits viral RNA-dependent RNA polymerases, causing chain termination and lethal mutagenesis [40,41]. Unlike remdesivir, favipiravir can be taken orally, making early outpatient treatment feasible. Favipiravir exhibits antiviral activity towards various RNA viruses, including influenza [37], Ebola [42], and other emerging pathogens [43,44], and has proven effective in the reduction of SARS-CoV-2 replication in vitro and in vivo [45,46]. In studies involving favipiravir treatment of patients with COVID-19, the results have been inconsistent, with some reports showing a positive impact on viral load and symptom duration and others indicating a low efficacy [45,47,48]. This variability likely reflects differences in the dosing schedules, treatment timing, and patient demographics, and suggests that the optimal use of favipiravir against SARS-CoV-2 is not yet well defined.

Mathematical models of viral dynamics provide a quantitative framework for studying infection kinetics and can be used to help optimize treatment regimens. These models typically use ordinary differential equation based systems that describe how infection takes place within a population of target cells, infected cells, and viruses [49,50]. They can also be used to obtain estimates of various biological parameters [50], allowing for the characterization and quantitative comparison of viral infections. Crucially, these models can reveal which biological parameters a drug affects [51,52,53], information that viral load trajectories alone cannot necessarily provide. Models for viral dynamics have been successfully used to characterize the antiviral treatment of a number of viral infections, including hepatitis C virus [53], HIV [54], influenza [55,56], respiratory syncytial virus [52], Zika [57], and Ebola [58,59]. The parameterization of the mathematical models and the characterization of the drug effect allow for the use of the models to predict and optimize treatment regimens [57,60,61], including predicting the effect of combination therapy [62,63,64], the effectiveness of treatment during viral coinfections [65], or the effect of drug pressure on viral evolution [66,67].

In this paper, we fit a target-cell-limited ordinary differential equation model independently to time series viral load data from each of 30 individual cynomolgus macaques infected with SARS-CoV-2 and treated with favipiravir at three different doses (100 mg/kg, 150 mg/kg, and 180 mg/kg) as described by Marlin et al. [68]. We found no statistically significant differences in any of the model parameters between the control and any of the treatment groups. A subsequent simulation analysis of the statistical power of the experimental study suggested that favipiravir would need to reduce the viral production by more than 90% in order to be detected, given the small sample sizes. Fitting the same model to the group-averaged viral loads, however, resulted in findings of statistically significant differences in the viral production rate, infectious-cell lifespan, and viral clearance rate. This highlights differences in the analysis results when using individual data versus averaged group data that removes animal-to-animal variation, particularly when there is substantial variability in individual time courses.

## 2. Materials and Methods

### 2.1. Experimental Data

This analysis employed longitudinal SARS-CoV-2 viral load data from cynomolgus macaques as described in Marlin et al. (2022) [68]. Animals were allocated to one of four experimental groups: an untreated control group (n=10) and three favipiravir-treated groups receiving 100 mg/kg (n=5), 150 mg/kg (n=10), or 180 mg/kg (n=5) twice daily. All treated animals were given a loading dose of 200 mg/kg and were given maintenance doses at the indicated concentrations. Two days after the initiation of treatment, the animals were exposed to $10^{6}$ pfu of the hCoV-19/France/lDF0372/2020 strain of SARS-CoV-2 via a combined intranasal and intratracheal route. Nasopharyngeal swabs were taken daily for 7 days.

### 2.2. Mathematical Model

The viral dynamics were modeled using a standard target-cell-limited model consisting of uninfected target cells (*T*), infected cells in an eclipse phase (*E*), productively infected cells (*I*), and free virus (*V*) [69]. The system of ordinary differential equations is given by

(1) $$\begin{array}{cc} \frac{dT}{dt} & =-\beta TV,\\ \frac{dE}{dt} & =\beta TV-kE,\\ \frac{dI}{dt} & =kE-\delta I,\\ \frac{dV}{dt} & =pI-cV. \end{array}$$

The equations include β, the rate of target cell infection; *k*, the rate at which cells transition from the eclipse phase to fully infected; δ, the rate of removal of infected cells; *p*, the rate of new virus production; and *c*, the clearance rate of virions.

### 2.3. Data Fitting

The model parameters were estimated independently for each of the 30 individual animals by fitting the viral dynamics model to the observed time series of the $\log_{10}$ viral titer. Parameter estimation was performed by minimizing the sum of squared residuals (SSR) between the observed and predicted viral loads:

(2) $$\mathrm{SSR}=\sum_{i=1}^{n}{\left(\log_{10}\left(V_{\mathrm{data},i}\right)-\log_{10}\left(V_{\mathrm{model},i}\right)\right)}^{2},$$

where $V_{\mathrm{data},i}$ denotes the measured viral load at time point *i* and $V_{\mathrm{model},i}$ denotes the corresponding model prediction.

Optimization was carried out via the scipy.minimize function using the Nelder–Mead algorithm, followed by a bounded refinement step using the L-BFGS-B algorithm. We used a relative cell count, so assumed that the initial target cell count was T(0)=1. We set V(0) to the initially measured viral load for each animal and E(0)=I(0)=0. Six initial parameter guesses were evaluated per animal, spanning a range of biologically plausible values, and the parameter set yielding the smallest SSR was selected as the best fit. All parameters were constrained to physically plausible ranges during optimization, as summarized in Table 1.

### 2.4. Statistical Analysis

The parameter uncertainty was assessed using a nonparametric residual bootstrap procedure applied independently to each animal. For each animal, 1000 surrogate datasets were generated by resampling the residuals from the best-fit model and adding them to the model-predicted trajectory. The viral dynamics model was then re-fit to each surrogate dataset using the same fitting procedure as described above, yielding 1000 bootstrap parameter estimates per animal.

To quantify the parameter uncertainty, the bootstrap distributions from all animals within a treatment group were pooled into a single group-level distribution for each parameter, and 95% confidence intervals were estimated as the 2.5th and 97.5th percentiles of the pooled within-group bootstrap distribution. Because pooling bootstrap replicates from multiple animals conflates the estimation uncertainty with biological variability, the bootstrap distributions were used solely for confidence interval estimation and were not used as the basis for hypothesis testing.

Statistical comparisons between the control group and each favipiravir-treated group were performed using two-sided Mann–Whitney U tests applied directly to the per-animal best-fit parameter estimates, treating each animal as an independent biological observation. Drug-versus-drug comparisons (e.g., 100 mg/kg vs. 150 mg/kg) were not performed, as the primary scientific question concerns whether treatment with favipiravir differs from the untreated control. In total, fifteen tests were performed (five parameters × three dose groups); no correction for multiple comparisons was applied because all tests were pre-specified and hypothesis-driven, and none approached statistical significance.

To assess the statistical power available to detect treatment effects at the observed group sizes, we conducted a simulation-based power analysis targeting the viral production rate *p*. For each combination of group size (n=5 or n=10) and true effect size (10–99% reduction in *p*), we generated synthetic datasets by drawing parameter sets from the empirical control group distribution, reducing *p* by the specified percentage, simulating viral trajectories with additive log-normal noise (SD = 0.40 log_10_ units, approximating the median root-mean-square residual across the individual animal fits), and re-fitting the ODE model to recover the per-animal estimates. The proportion of trials (out of 100) in which the two-sided Mann–Whitney U test reached α=0.05 was recorded as the estimated power.

## 3. Results

### 3.1. Model Fits to Individual Animals

The viral dynamics model was fit independently to data from each of the 30 animals. The best-fit trajectories for each group are shown in Figure 1, Figure 2, Figure 3 and Figure 4, with each panel displaying one animal and its SSR. The median parameter estimates per group with 95% bootstrap confidence intervals are given in Table 2 and summarized in Figure 5. Corner plots for all the model fits are included in the Supplementary Material. For the majority of animals, the model captured the qualitative features of the viral time course, including the initial expansion and subsequent clearance. Examples of viral titer data with particularly good model fits are MF22 (SSR = 0.02), MF36 (SSR = 0.08), MF25 (SSR = 0.22) and MF35 (SSR = 0.39), while MF30 (SSR = 8.54), MF40 (SSR = 10.96) and MF42 (SSR = 12.47) did not show a close agreement between the model and the data, resulting in substantially larger residuals. We note that the poorest fits occurred in animals whose viral load time series did not follow the smooth unimodal peak and decline typically predicted by the target-cell-limited model.

The parameter estimates varied substantially across animals, both within and between treatment groups. The median infection rate β was $2.4\times 10^{-7}$ mL copies^−1^ d^−1^ in the control group and ranged from $4.9\times 10^{-7}$ to $2.6\times 10^{-6}$ mL copies^−1^ d^−1^ in the treated groups, with the confidence intervals for all groups spanning several orders of magnitude. The median eclipse transition rate *k* was higher in the control group (18.5 d^−1^) than in the treated groups (1.7–3.5 d^−1^). The infected-cell loss rate δ, viral production rate *p*, and viral clearance rate *c* showed no consistent directional pattern across dose levels. Notably, the bootstrap confidence intervals for *k* and β extended to or near the upper parameter bounds in all groups, indicating that these parameters are not practically identifiable with the available data. Note that the pooled confidence intervals reflect both the per-animal estimation uncertainty and the between-animal variability within each group. The estimated infected-cell death rate δ (median: 1.27 d^−1^, corresponding to an infected-cell lifespan of approximately 19 h) was consistent with values reported in previous SARS-CoV-2 target-cell-limited modeling studies. Gonçalves et al. fitted the same model structure to nasopharyngeal viral load data from cynomolgus macaques and reported δ=1.9 d^−1^ [70]; Ke et al. reported δ=1.7 d^−1^ in a human cohort [71]; and Zhang et al. estimated δ=1.04 d^−1^ [72]. The median eclipse transition rate *k* in the control group (18.5 d^−1^) was higher than the values of 3–4 d^−1^ typically reported or assumed in other studies [70,71,72], but given that the bootstrap confidence interval for *k* extended to the upper parameter bound in all groups, this discrepancy likely reflects parameter non-identifiability rather than a genuine biological difference. A direct comparison of β and *p* across studies is not straightforward because these parameters depend on the measurement units of the viral titer, which are not absolute and can depend on environmental conditions in the lab [73].

### 3.2. Effect of Favipiravir Treatment

The mean *p*-values from the Mann–Whitney U tests comparing the control group to each favipiravir dose group are reported in Table 3. Given the mechanism of action of favipiravir, we expected to observe differences in the viral production rate between treated and untreated groups. No comparison reached statistical significance for any parameter (all p>0.06). The smallest *p*-values were observed for *k* (control vs. 150 mg/kg: p=0.064) and *c* (control vs. 150 mg/kg: p=0.076; control vs. 180 mg/kg: p=0.099), but none approached the α=0.05 threshold.

Given our inability to detect a treatment effect with favipiravir for any treatment group, we explored whether the negative result could be due to the small number of animals in each treatment group. We performed a simulation-based power analysis to determine whether the number of animals used in the study was limiting our ability to detect the effect of favipiravir. We assumed that favipiravir lowered the production rate, then used control group parameter sets to generate both control and synthetic treatment datasets with a specified percentage reduction in *p*. The Mann–Whitney U test was applied to the resulting per-animal estimates (Figure 6) for group sizes of five animals (orange line) or ten animals (blue line). The simulations showed that a group size of ten animals had 80% power when *p* was reduced by ∼94% and a group size of 5 animals did not rise to that level, even when *p* was reduced by 99%. Thus, the original study was only capable of detecting favipiravir if the antiviral caused a very large reduction in the viral production rate.

### 3.3. Fitting Averaged Viral Titer Data

Individual viral titers are often not included in published studies, particularly for human clinical trials. Instead, the results are often presented as averaged viral titer time courses for each treatment group. Given that this single averaged curve is often all that is available to mathematical modelers, we also carried out the fitting and the bootstrap using a group-level approach, whereby the data were first averaged among the animals in each group before the fitting was done. On each sample day, the viral loads for all the animals in a group were averaged together using the geometric mean (or the arithmetic mean of the $\log_{10}$ viral loads), resulting in one averaged viral load curve per group. The target-cell-limited model, Equation (1), was fitted to each group-averaged curve once, using the same optimization approach described in the Methods (Section 2.3). The bootstrap, with 1000 replications, was performed on each group-averaged curve.

The best-fit trajectories for the four group-averaged datasets are shown in Figure 7, and the corresponding parameter estimates with 95% bootstrap confidence intervals are given in Table 4. Corner plots for the group-averaged fits are included in the Supplementary Material. The model fit well with the average trajectories in the 100 mg/kg and 180 mg/kg groups (SSR = 0.106 and 0.223, respectively), but not as well with the control and 150 mg/kg groups (SSR = 0.349 and 0.996, respectively), showing that there are more day-to-day fluctuations once the viral titers from individual animals are combined. Averaging across animals also shifts the parameter estimates substantially relative to the individual-level medians in Table 2: the control group’s viral production rate is estimated here at $p=2.04\times 10^{9}$ copies mL^−1^ cell^−1^ d^−1^, roughly double the individual-level median of $1.06\times 10^{9}$, while the control infection rate is estimated at $\beta =1.94\times 10^{-6}$ mL copies^−1^ d^−1^, roughly an order of magnitude larger than the individual-level median of $2.4\times 10^{-7}$. Both analyses agree qualitatively that the treated groups had a lower *p* than the control, but the apparent direction of differences in other parameters, as well as the magnitude and precision of the estimated effects, depended heavily on which analysis strategy was used.

The bootstrap parameter distributions for the four groups obtained from this averaged-data approach are shown in Figure 8. In marked contrast to the individual-level bootstrap distributions in Figure 5, which span several orders of magnitude within a single treatment group, the group-averaged bootstrap distributions were comparatively narrow and well separated between the groups for several parameters, most notably *p* and β. This apparent precision arises because bootstrapping a single averaged trajectory captures only the uncertainty in fitting that one curve, and cannot reproduce the biological variability between individual animals. Consequently, these distributions should not be interpreted as evidence of statistically significant treatment differences; they reflect the model fit uncertainty under an averaging assumption, not the true between-animal biological spread.

As an illustration of the potentially misleading conclusions that can be drawn from averaged data, we used the Mann–Whitney U test to compare the control group to each favipiravir dose group. The resulting *p*-values are given in Table 5. Unlike the individual-level comparisons in Table 3, where no parameter reached statistical significance in any comparison, the averaged-data approach identified several apparent differences: the viral production rate *p* was lower in the 100 mg/kg and 150 mg/kg groups relative to the control (mean p=0.003 for both), the infected-cell loss rate δ differed between the control and both the 100 mg/kg (mean p=0.012) and 150 mg/kg (mean p=0.034) groups, and the viral clearance rate *c* differed between the control and the 180 mg/kg group (mean p=0.035).

## 4. Discussion

We fit a target-cell-limited viral dynamics model independently to longitudinal SARS-CoV-2 viral load data from each of 30 individual cynomolgus macaques treated with favipiravir at four doses (0, 100, 150, and 180 mg/kg), and used bootstrap resampling to quantify the parameter uncertainty at the individual level. The primary finding is that no statistically significant differences in any model parameter were detected between the untreated control group and any favipiravir dose group. These results differ from what might be expected given the known mechanism of favipiravir, which inhibits viral RNA-dependent RNA polymerase. This inhibition appears to lead to both chain termination and lethal mutagenesis that result in a reduced production of viable virions [74]. Previous in vitro and animal studies have found that favipiravir reduces the viral titer and viral replication rate of SARS-CoV-2 [43,75], which would be expected to appear as a reduced viral production rate *p* or increased viral clearance rate *c* in the model.

Notably, we found both a reduced production rate and an increased viral clearance rate, although not consistently for all favipiravir doses, when fitting the model to group-averaged viral titer data. However, we also found a reduced infectious-cell death rate, which would not be consistent with an effective antiviral. Several factors likely contributed to these patterns. First, the 180 mg/kg group contained only five animals, two of which had truncated viral load time series, meaning the averaged trajectory for that group reflects a small subset of animals rather than a representative dose-group response, with the viral decay portion of the viral titer curve being most strongly affected by the missing data. Second, the between-animal heterogeneity within each group means that the shape of any averaged trajectory depends strongly on the specific animals and how their individual time courses happen to align. Thus, fitting a single deterministic model to an averaged curve can produce parameter estimates that reflect the mathematical requirements of fitting that particular curve shape rather than any biologically meaningful trend. Averaging suppresses natural variability between animals prior to its propagation into bootstrap distributions, leading to statistical comparisons on averaged data that can be more precise than the biology warrants. Our simulation-based assessment of the power of the original study suggests that a group size of five animals is unable to detect even a nearly 100% effective viral production block by favipiravir, suggesting high inter-animal variability in the response that is removed when averaging. A previous study examining the oseltamivir treatment of influenza also compared individual model fits to group-averaged fits and found that the analysis using individual fits showed a less-definitive benefit of oseltamivir treatment than the group-averaged analysis [76]. Together, these studies suggest that median viral titer curves should be used with caution, as they are likely not representative of the viral time course in an individual and do not capture the full variability in individual time courses.

There are a number of biological reasons for individual variability in the infection time course of SARS-CoV-2. Most notably, there is heterogeneity in individual host immune responses that lead to differences in the severity of the resulting disease [77]. In particular, an impaired interferon response has been linked to severe disease [78], with some of these differential IFN responses linked to genetic variation in the patients [79]. The adaptive immune response also shows distinct phenotypes in both antibody and T cell responses that lead to different infection time courses [80]. These differences could be driven, in part, by differences in the recognition of SARS-CoV-2 antigens [81]. Another important factor affecting the time course of SARS-CoV-2 infection is the expression of the ACE2 receptor, which has been shown to vary considerably in humans [82,83], with this variability being linked to variation in the disease severity [84,85]. The individual pharmacokinetic response to favipiravir can also vary substantially [86], with some patients not maintaining the minimal expected plasma concentrations of the drug when given the standard dose [87]. Finally, given that favipiravir can cause viral mutagenesis [88], viral evolution during treatment can also add heterogeneity to the time course of the infection depending on the fitness and antiviral susceptibility of the viral mutations that appear. This heterogeneity also has practical implications for antiviral dosing strategies: if individual kinetics vary as broadly as observed here, group-averaged analyses will systematically underestimate the variability in the treatment response, and population-level dosing recommendations derived from such analyses might be poorly calibrated for a substantial fraction of individuals.

The absence of a detectable signal at the individual animal level does not necessarily mean that favipiravir is ineffective; rather, an effect may exist, but it is smaller than the between-animal variability in this cohort. Within the control group alone, individual estimates of β span more than five orders of magnitude and estimates of *k* are largely unconstrained above a lower bound. There are two sources for this kind of variability: biological variation between animals in the viral dynamics, and the fact that the target-cell-limited model is not identifiable in short time series [89,90,91,92,93]. Because the within-group spread in the parameter estimates is large, the sample sizes available (5–10 animals per group) provide limited statistical power to detect differences between groups. Even if real differences exist between the treatment groups, our simulations suggest that this would only be detected if they resulted in well over a 90% reduction in viral production. There are several changes to the experimental protocol that could help potentially improve parameter identifiability. In addition to more frequent sampling of the viral load, measuring the number of infected cells, perhaps through a fluorescently labeled virus [94,95], would provide additional measurements of a second model variable that would help improve parameter identifiability. Measuring both the infectious virus and viral RNA has also been shown to improve the identifiability of model parameters [96]. To address the issue of limited data on the modeling side, fixing the values of some parameters can improve the identifiability of the remaining parameters [89,92], provided there are well-known values for the fixed parameters. For viral dynamics models, *c*, *k*, or δ are often fixed, since independent in vitro experiments can be done to estimate values for these quantities [55,97,98,99]. However, it is not clear that the in vitro values, particularly for viral clearance (*c*) and infectious-cell clearance (δ), translate directly to in vivo systems where there are immune responses that contribute to the clearance of both the virus and infected cells, resulting in higher values of *c* and δ than would be measured in vitro. Additionally, other approaches to fitting, such as Bayesian or hierarchical approaches, could provide tighter bounds on some of the identifiable parameters or at least more clearly separate biological variability from parameter uncertainty [100,101].

Other studies on the effectiveness of favipiravir in SARS-CoV-2 patients are also mixed. Several studies have shown no benefit when favipiravir was added to the standard of care for hospitalized, severely ill patients [102,103,104,105,106,107,108,109]. However, other studies suggest that there might be some benefit, particularly in patients with more mild illness. A small study found that favipiravir could help terminate SARS-CoV-2 shedding in patients who had prolonged viremia [110], with other studies showing that favipiravir lowered the time to symptom resolution [107,108,111,112,113,114]. It appears that favipiravir might also help reduce the transmission of SARS-CoV-2 [115], so it might be useful in helping to stem the spread of SARS-CoV-2. There is still interest in exploring the use of favipiravir in combination with other antivirals [116,117,118], as it shows pharmacokinetic interactions with some other antivirals [119]. There are also efforts to develop new antivirals using favipiravir as a base [120,121] or to improve the efficacy of favipiravir using nanoparticle-based drug delivery methods [122,123,124].

There are a number of limitations in the experimental design of the data used in this experiment. As noted earlier, the number of animals in each group was rather low, only five to ten animals, and based on our simulation study, it was not sufficient to detect small changes in parameter values caused by favipiravir. Furthermore, the viral load was measured using nasopharyngeal swabs, while the replication of SARS-CoV-2 takes place primarily in the lower respiratory tract. Viral RNA at the mucosa may not reflect the processes occurring in the primary focus of infection [125]. In addition, the animals were infected with the virus using the intranasal and intratracheal routes, which does not quite coincide with natural human infection. Notably, the route of infection can also change the underlying viral kinetics [126]. Finally, while cynomolgus macaques appear to have similar infection trajectories to mild cases of SARS-CoV-2 in humans [127], there are physiological differences between the species that could result in differences in the treatment outcomes.

The model is also very simple in that it does not consider the immune response, although both the innate and adaptive immune systems participate in controlling the infection of SARS-CoV-2 in vivo [127]. Thus, all the mechanisms that deal with eliminating infected cells, such as natural cell death and immune-cell-mediated elimination [128], are incorporated into one parameter, δ. Similarly, the viral clearance rate includes immune responses such as the effect of antibodies [129]. The model also does not include biologically realistic transitions from the eclipse to infectious states [130,131] or from infectious to dead [132]. It also assumes that viral production is constant over the infectious lifespan of the cell, which single-cell viral production experiments have shown is not the case [133] and could result in differences in the model time course predictions [134]. Finally, the pharmacokinetics of favipiravir were not considered [135], since our study assumed that favipiravir resulted in a constant change to the model parameters. A previous modeling study showed that the assumption of a constant drug effect can replicate the viral titer curves produced by a pharmacokinetically realistic drug effect [136]. In particular, the study showed that a viral titer curve produced with a constant drug assumption and one produced with a realistic dosing regimen differed substantially when the drug dose was high (>50 times the IC_50_) and the dosing interval was large (>36 h). Since [136] used oseltamivir treatment of influenza as an example, the results will not be exactly the same for the favipiravir treatment of SARS-CoV-2, however, the dose and dosing interval for favipiravir used in this study should be in the range where a constant drug effect is a reasonable approximation. Additionally, plasma measurements of favipiravir concentrations in macaques were fairly stable over 14 days with the BID regimen used in the study (Figure 2 of [68]), further supporting the assumption of a constant drug effect. Such detailed refinements to the model will require a more comprehensive dataset in order to ensure parameter identifiability.

## 5. Conclusions

We fit a target-cell-limited viral dynamics model to SARS-CoV-2 viral load data from 30 individual cynomolgus macaques across four treatment groups (untreated control and three favipiravir dose levels), and used residual bootstrap resampling to generate per-animal parameter distributions that were pooled by group for statistical comparison. Individual-level fitting revealed substantial inter-animal variability in all five model parameters, with the bootstrap confidence intervals spanning several orders of magnitude for most parameters and groups. Mann–Whitney U tests comparing the control to each treated group found no statistically significant differences in any parameter at any dose level. These results indicate that, at the cohort sizes used in this study, an individual-level analysis does not provide sufficient statistical power to resolve favipiravir-induced changes in the viral kinetics parameters. Future studies should consider larger cohorts, more frequent viral sampling, and explicit pharmacokinetic modeling to improve the identifiability of treatment effects at the individual animal level.

## Figures and Tables

**Figure 1:**
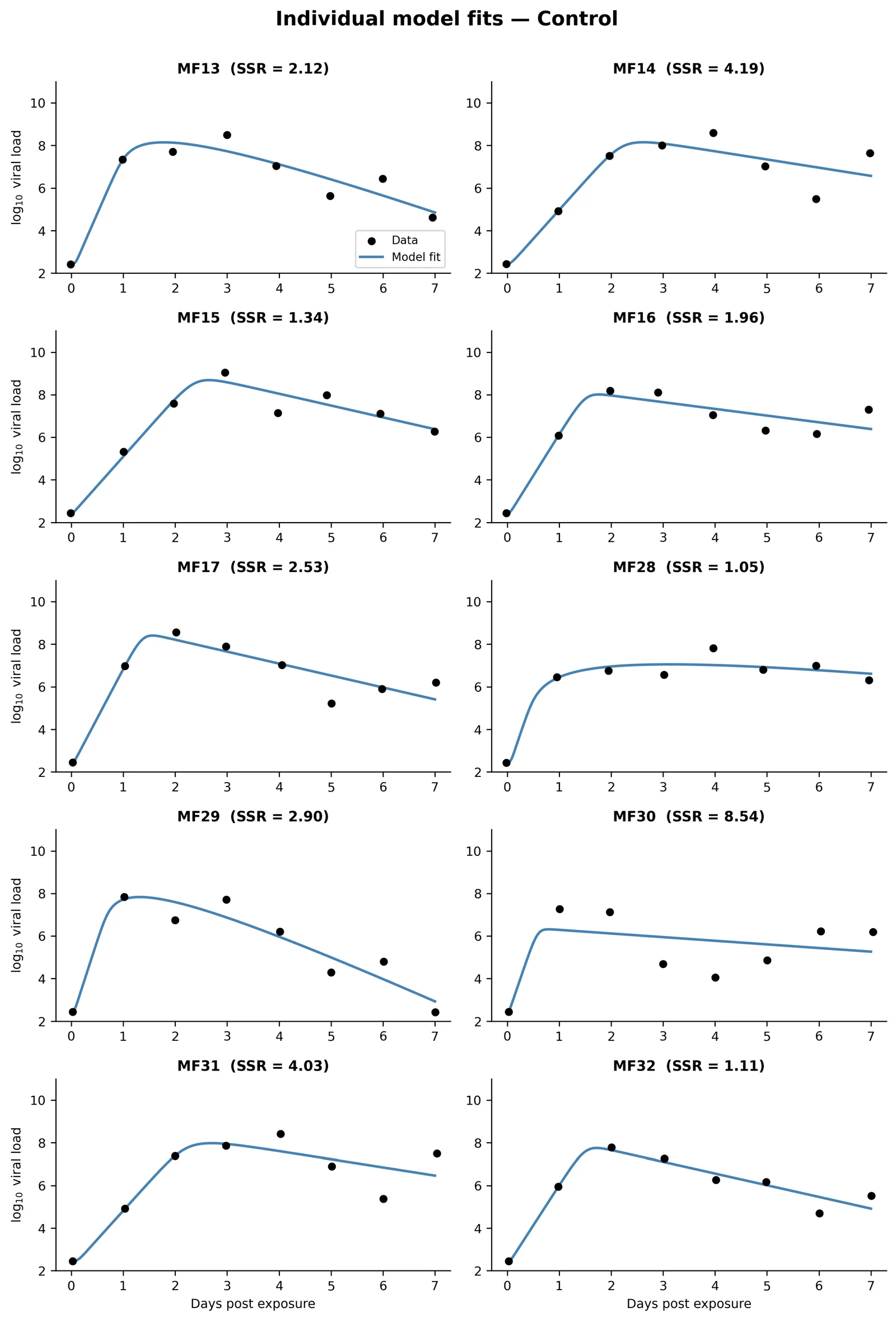
Individual best-fit model trajectories for the 10 control animals. Points represent the observed $\log_{10}$ viral load; curves represent the model fits. Each panel title gives the animal identifier and SSR.

**Figure 2:**
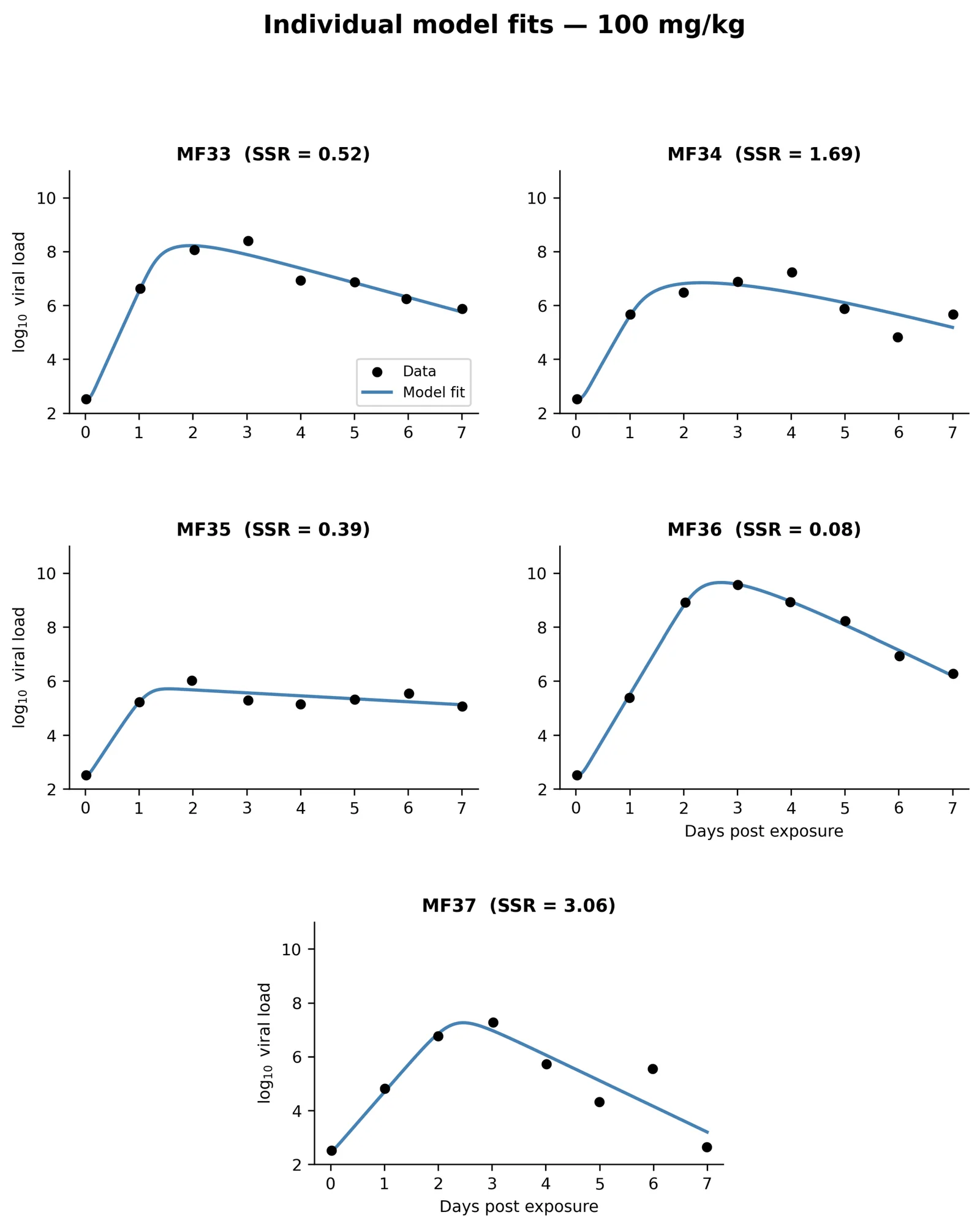
Individual best-fit model trajectories for the five animals in the 100 mg/kg favipiravir group. Points represent the observed $\log_{10}$ viral load; curves represent the model fits. Each panel title gives the animal identifier and SSR.

**Figure 3:**
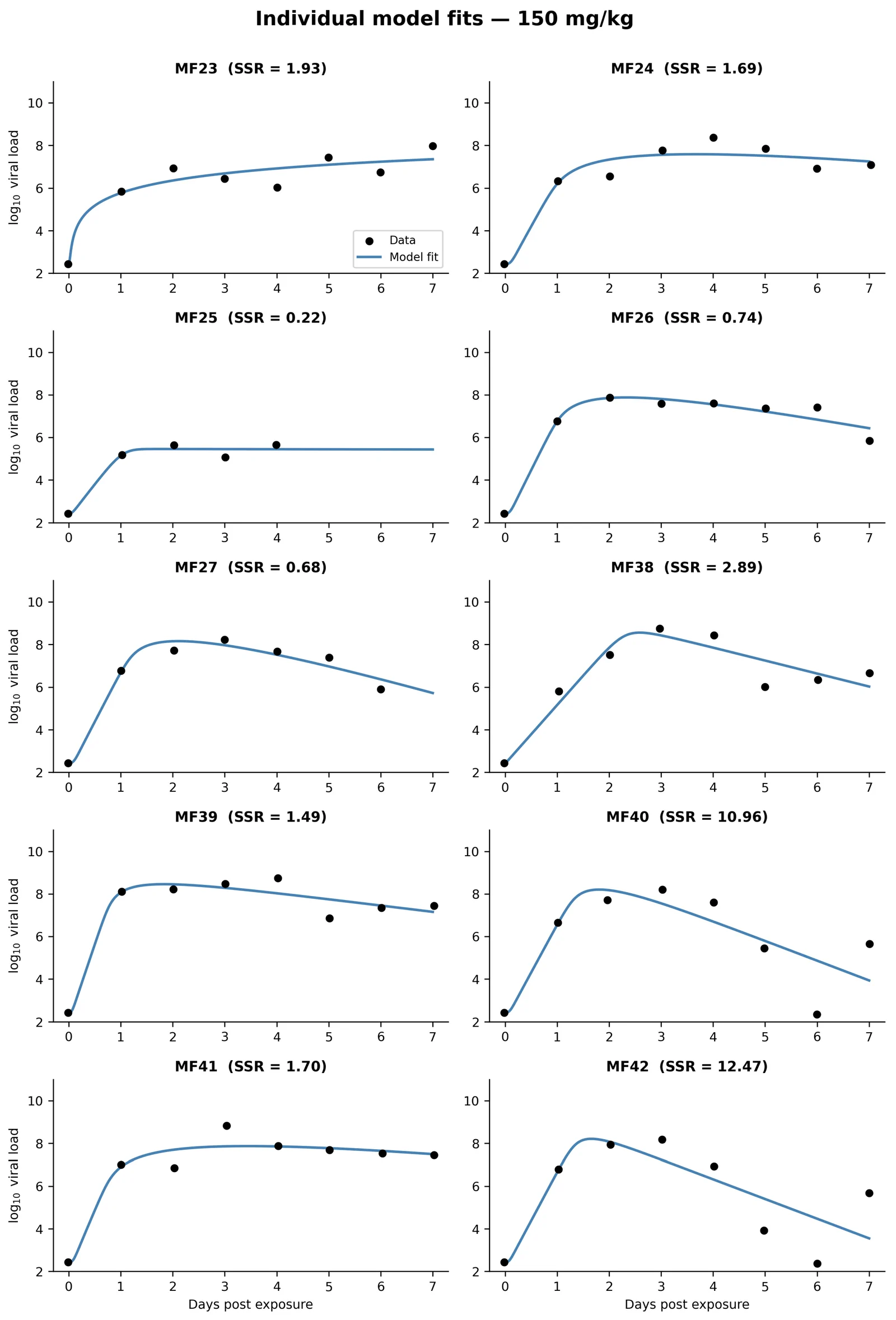
Individual best-fit model trajectories for the 10 animals in the 150 mg/kg favipiravir group. Points represent the observed $\log_{10}$ viral load; curves represent the model fits. Each panel title gives the animal identifier and SSR.

**Figure 4:**
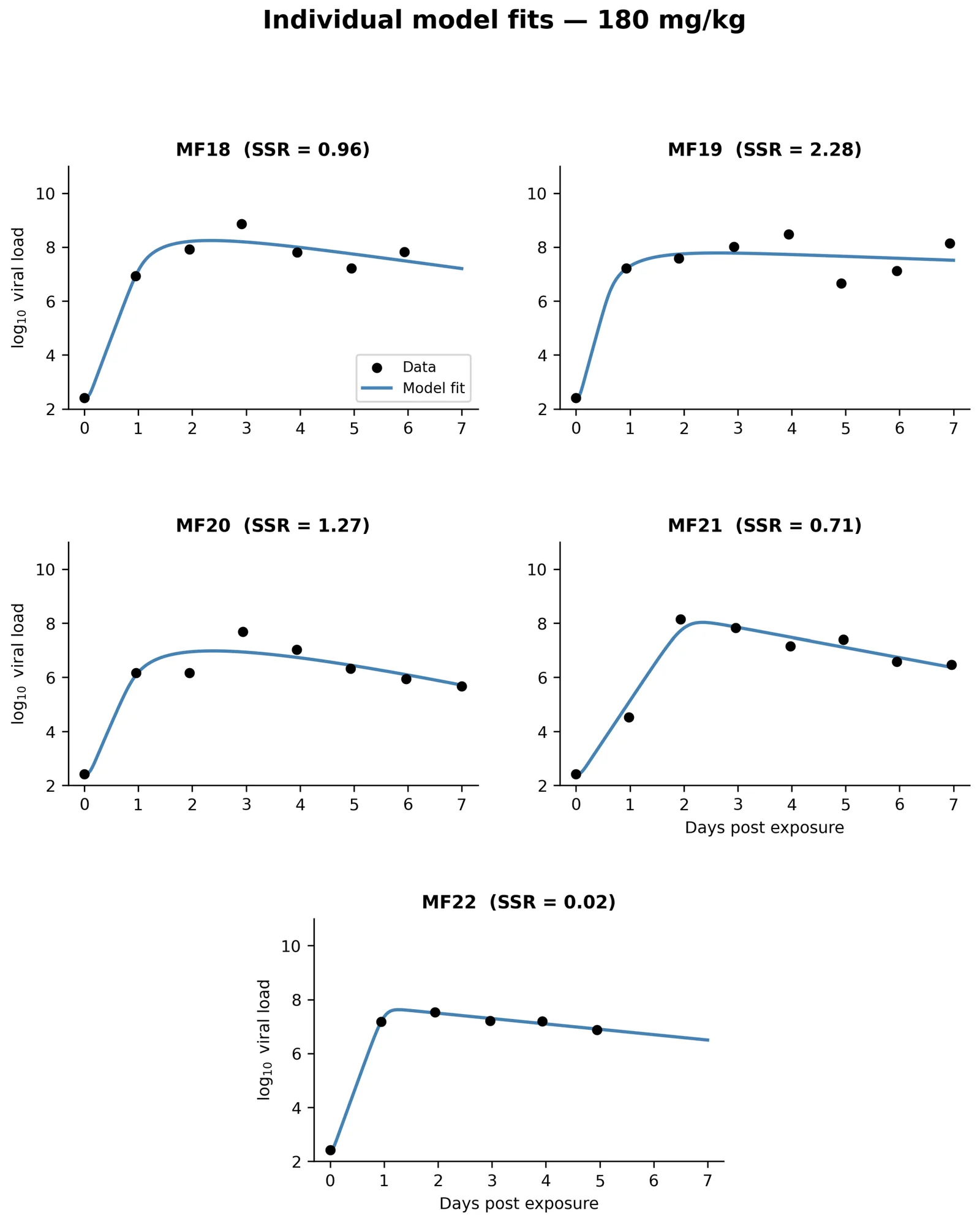
Individual best-fit model trajectories for the five animals in the 180 mg/kg favipiravir group. Points represent the observed $\log_{10}$ viral load; curves represent the model fits. Each panel title gives the animal identifier and SSR.

**Figure 5:**
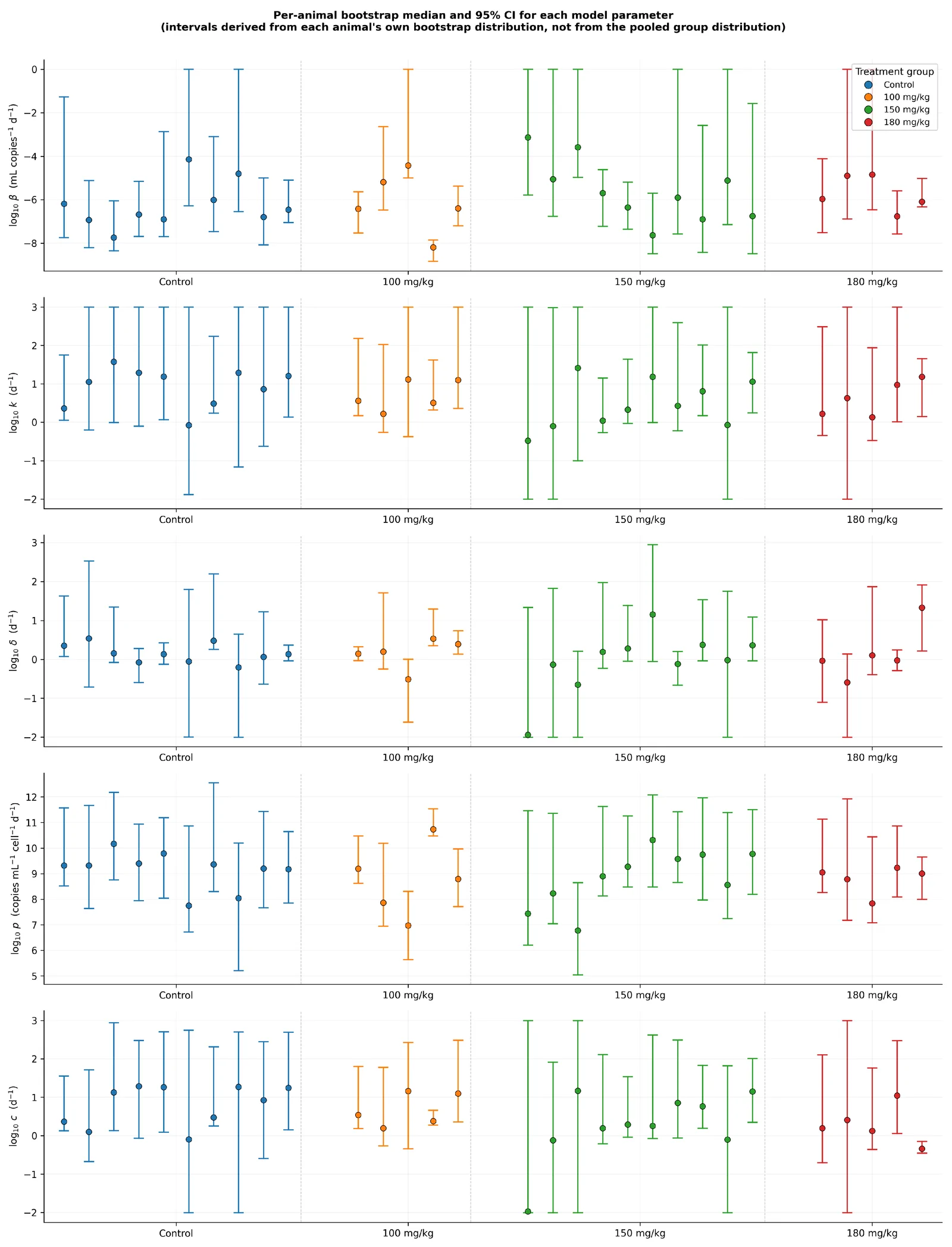
Summary of median parameter estimates from individual animal fits with 95% confidence intervals.

**Figure 6:**
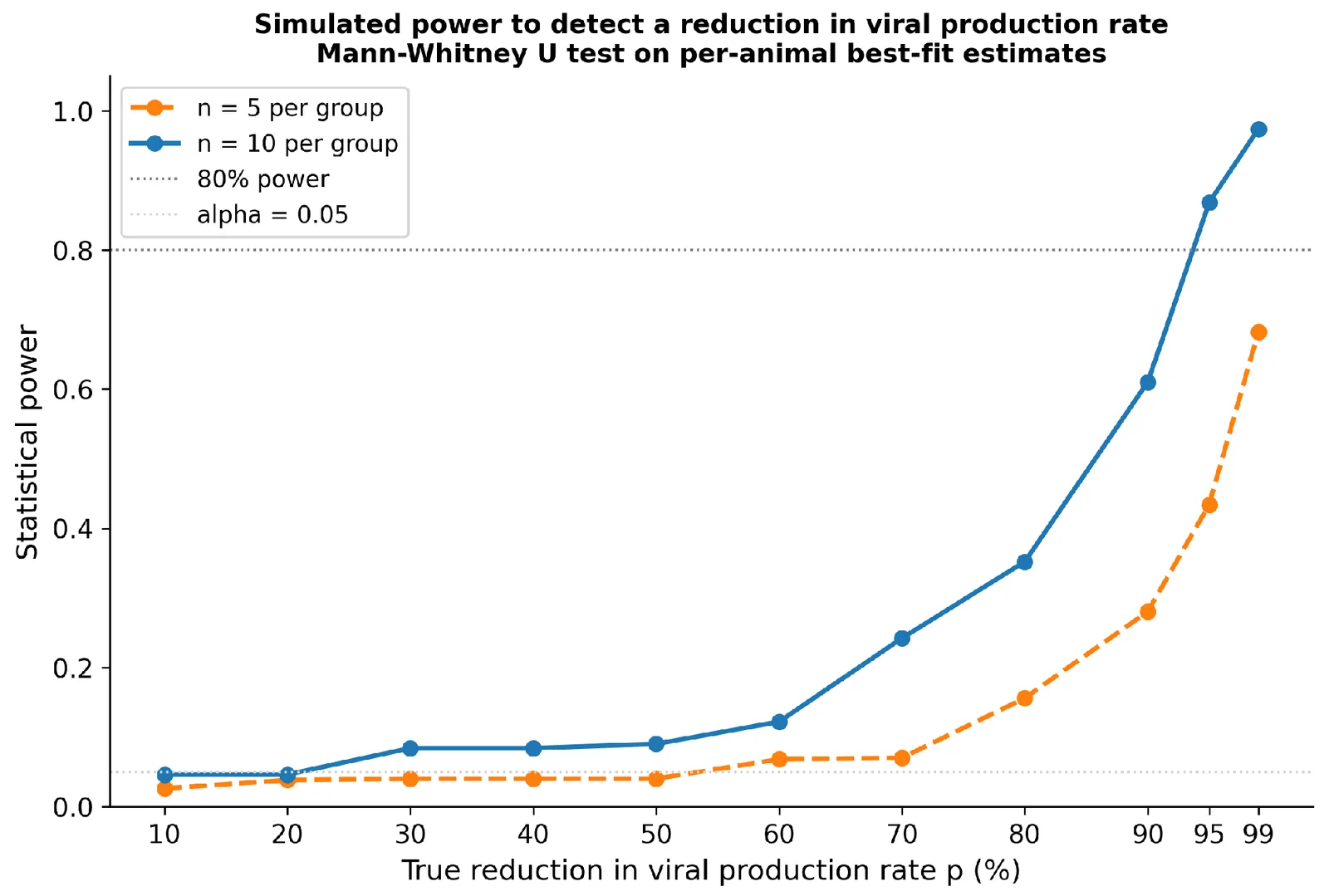
Simulation-based statistical power to detect a true reduction in the viral production rate *p* as a function of the effect size and sample size. Each point represents the proportion of simulated trials in which a two-sided Mann–Whitney U test on the per-animal best-fit *p* estimates reached α=0.05 (100 replicates for n=5; 50 replicates for n=10). The dashed horizontal line marks 80% power.

**Figure 7:**
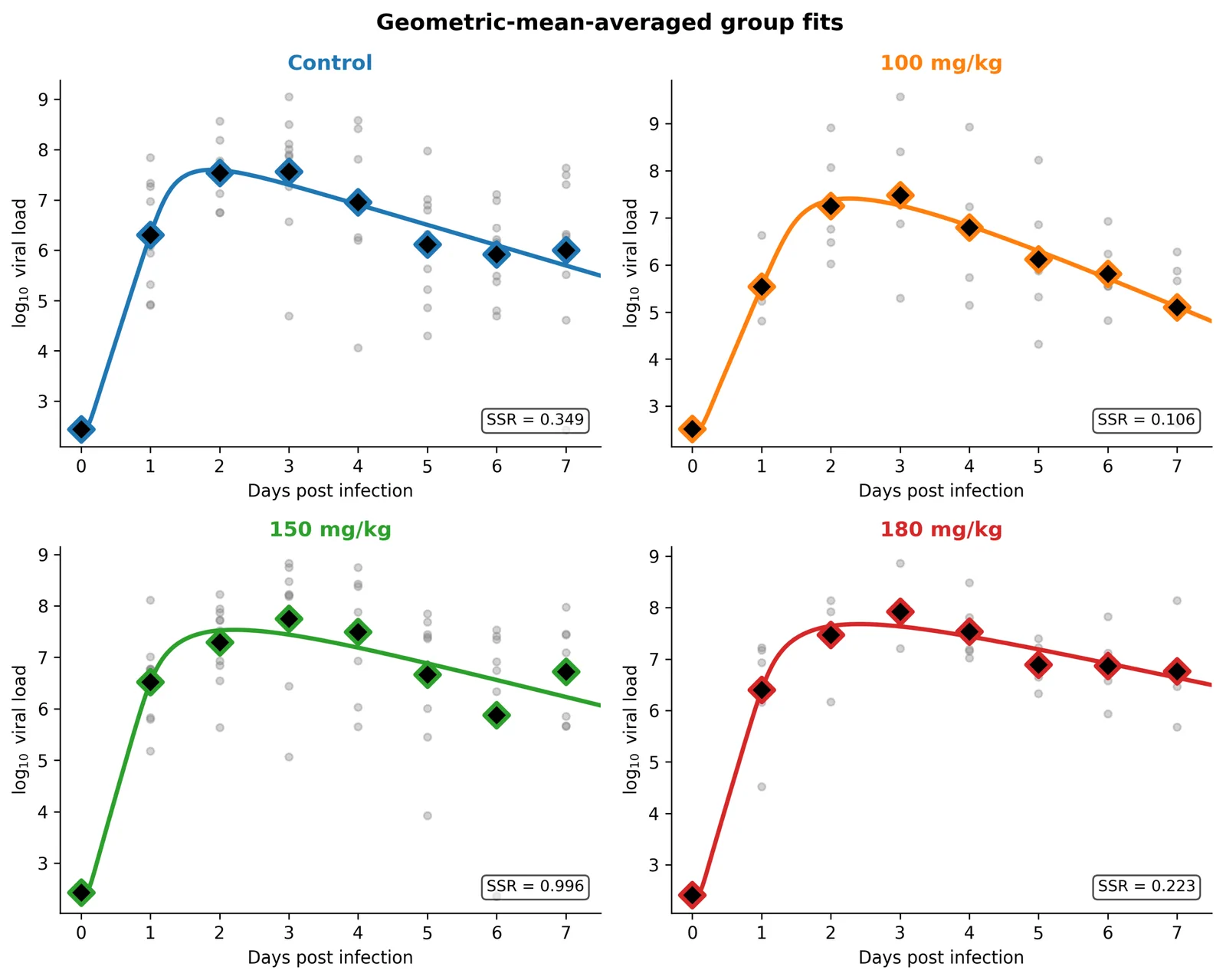
Best-fit model trajectories obtained by fitting once to the geometric-mean-averaged viral load data for each treatment group, rather than to each animal individually. Points represent the averaged $\log_{10}$ viral load at each sampling day; curves represent the group-level model fit; inset text gives the SSR of each fit.

**Figure 8:**
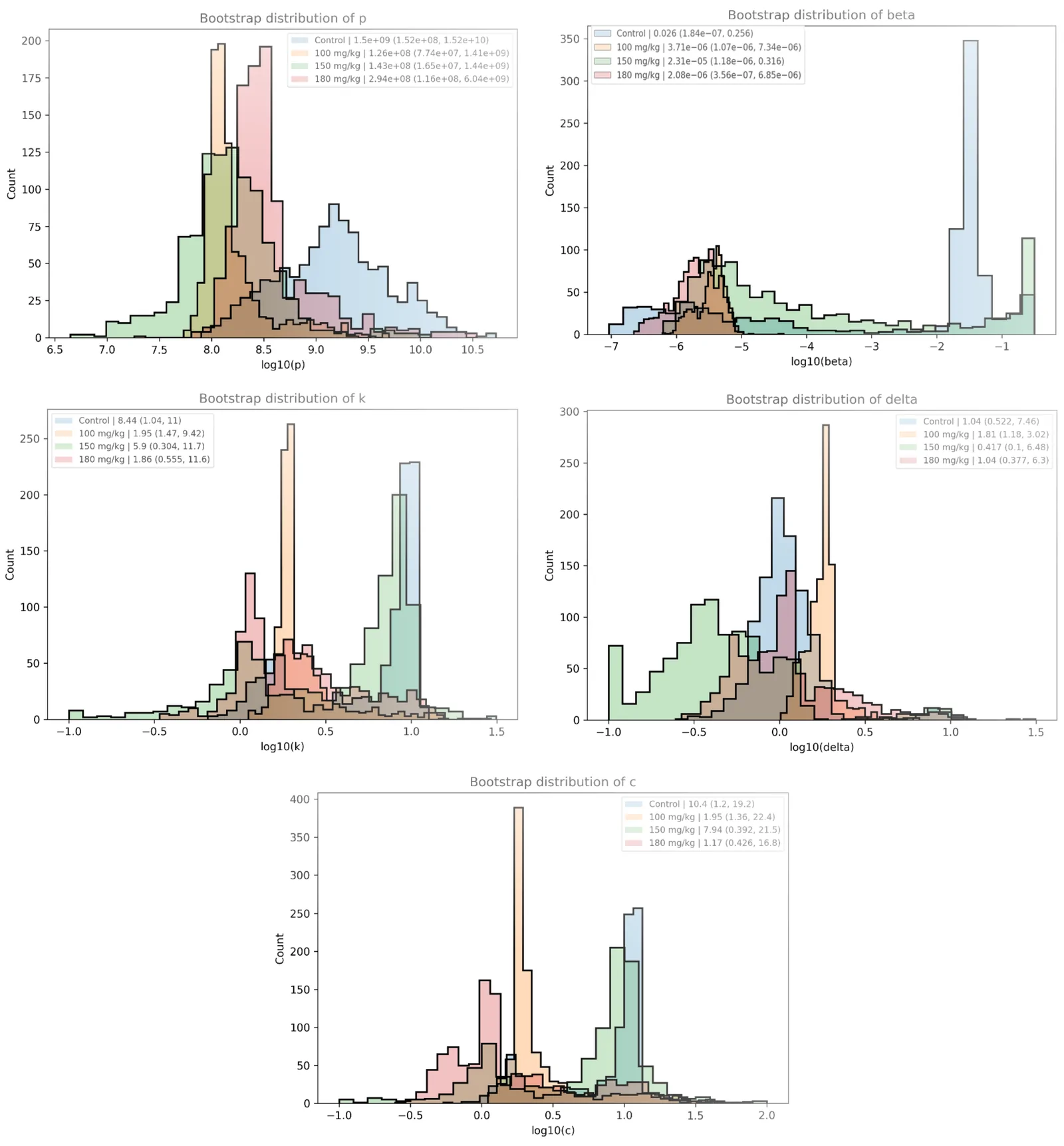
Bootstrap distributions of model parameters obtained by fitting to the geometric-mean-averaged group data (N=1000 replicates per group). Each panel shows the distribution for one parameter across the four treatment groups; legend entries give the median and 95% confidence interval for each group.

**Table 1:** Model parameters, biological descriptions, units, and bounds used during optimization.

Parameter	Description	Lower Bound	Upper Bound
β (mL copies^−1^ d^−1^)	Infection rate	$10^{-12}$	$10^{0}$
*k* (d^−1^)	Eclipse transition rate	$10^{-2}$	$10^{3}$
δ (d^−1^)	Infected-cell loss rate	$10^{-2}$	$10^{3}$
*p* (copies mL^−1^ cell^−1^ d^−1^)	Viral production rate	$10^{0}$	$10^{15}$
*c* (d^−1^)	Viral clearance rate	$10^{-2}$	$10^{3}$

**Table 2:** Median parameter estimates from individual animal fits with 95% bootstrap confidence intervals (2.5th–97.5th percentiles of the pooled within-group bootstrap distribution, N=1000 replicates per animal) for each treatment group.

Parameter	Control (n=10)	100 mg/kg (n=5)	150 mg/kg (n=10)	180 mg/kg (n=5)
β (mL copies^−1^ d^−1^)	$2.43\times 10^{-7}$	$4.93\times 10^{-7}$	$2.61\times 10^{-6}$	$1.89\times 10^{-6}$
95% CI	($8.6\times 10^{-9}$ to $9.0\times 10^{-1}$)	($2.7\times 10^{-9}$ to $2.1\times 10^{-3}$)	($6.1\times 10^{-9}$ to 1.0)	($3.9\times 10^{-8}$ to 1.0)
*k* (d^−1^)	18.5	3.47	1.72	2.01
95% CI	(0.37 to >999)	(0.68 to >999)	(0.01 to >999)	(0.39 to >999)
δ (d^−1^)	1.27	1.45	1.30	0.856
95% CI	(0.094 to 47.0)	(0.12 to 16.3)	(0.010 to 166)	(0.010 to 37.4)
*p* (copies mL^−1^ cell^−1^ d^−1^)	$1.06\times 10^{9}$	$5.88\times 10^{8}$	$7.74\times 10^{8}$	$6.96\times 10^{8}$
95% CI	($1.5\times 10^{7}$ to $3.2\times 10^{11}$)	($1.3\times 10^{6}$ to $1.4\times 10^{11}$)	($1.1\times 10^{6}$ to $4.5\times 10^{11}$)	($1.9\times 10^{7}$ to $5.7\times 10^{10}$)
*c* (d^−1^)	11.9	3.47	1.75	1.98
95% CI	(0.24 to 441)	(0.73 to 136)	(0.010 to 276)	(0.24 to 224)

**Table 3:** Mann–Whitney U test *p*-values comparing the control group to each favipiravir dose group. No comparison reached statistical significance (α=0.05).

Parameter	Comparison	Mean *p*-Value
β	Control vs. 100 mg/kg	0.768
	Control vs. 150 mg/kg	0.186
	Control vs. 180 mg/kg	0.310
*k*	Control vs. 100 mg/kg	0.513
	Control vs. 150 mg/kg	0.064
	Control vs. 180 mg/kg	0.099
δ	Control vs. 100 mg/kg	0.768
	Control vs. 150 mg/kg	0.678
	Control vs. 180 mg/kg	0.371
*p*	Control vs. 100 mg/kg	0.513
	Control vs. 150 mg/kg	0.571
	Control vs. 180 mg/kg	0.310
*c*	Control vs. 100 mg/kg	0.513
	Control vs. 150 mg/kg	0.076
	Control vs. 180 mg/kg	0.099

**Table 4:** Best-fit parameter estimates with 95% bootstrap confidence intervals (2.5th–97.5th percentiles, N=1000 replicates) obtained by fitting the model once per treatment group to the geometric-mean-averaged viral load trajectory, rather than to each animal individually as in Table 2.

Parameter	Control	100 mg/kg	150 mg/kg	180 mg/kg
β (mL copies^−1^ d^−1^)	$1.94\times 10^{-6}$	$2.72\times 10^{-6}$	$5.57\times 10^{-6}$	$4.74\times 10^{-6}$
95% CI	($1.6\times 10^{-7}$ to $1.1\times 10^{-5}$)	($6.6\times 10^{-7}$ to $4.4\times 10^{-6}$)	($1.8\times 10^{-7}$ to $3.8\times 10^{-4}$)	($2.3\times 10^{-7}$ to $1.4\times 10^{-5}$)
*k* (d^−1^)	3.82	2.29	1.94	1.74
95% CI	(1.18 to >999)	(1.47 to 25.4)	(0.61 to 331)	(0.73 to 45.1)
δ (d^−1^)	3.82	1.45	0.74	0.65
95% CI	(1.23 to >999)	(1.14 to 1.97)	(0.37 to 1.81)	(0.39 to 1.33)
*p* (copies mL^−1^ cell^−1^ d^−1^)	$2.77\times 10^{8}$	$1.63\times 10^{8}$	$1.87\times 10^{8}$	$2.09\times 10^{8}$
95% CI	($8.7\times 10^{7}$ to $1.1\times 10^{12}$)	($1.1\times 10^{8}$ to $5.1\times 10^{10}$)	($3.9\times 10^{7}$ to >$10^{14}$)	($1.0\times 10^{8}$ to >$10^{14}$)
*c* (d^−1^)	0.93	2.20	2.63	2.11
95% CI	(0.71 to 1.63)	(1.52 to 499)	(0.82 to >999)	(0.92 to >999)

**Table 5:** Mean Mann–Whitney U test *p*-values (averaged over 100 subsamples of n=10 drawn from the group-level bootstrap distributions built on geometric-mean-averaged data) comparing the control group to each favipiravir dose group. Bold entries indicate p<0.05.

Parameter	Comparison	Mean *p*-Value
β	Control vs. 100 mg/kg	0.105
	Control vs. 150 mg/kg	0.413
	Control vs. 180 mg/kg	0.061
*k*	Control vs. 100 mg/kg	0.146
	Control vs. 150 mg/kg	0.336
	Control vs. 180 mg/kg	0.155
δ	Control vs. 100 mg/kg	**0.012**
	Control vs. 150 mg/kg	**0.034**
	Control vs. 180 mg/kg	0.546
*p*	Control vs. 100 mg/kg	**0.003**
	Control vs. 150 mg/kg	**0.003**
	Control vs. 180 mg/kg	0.068
*c*	Control vs. 100 mg/kg	0.107
	Control vs. 150 mg/kg	0.389
	Control vs. 180 mg/kg	**0.035**

## Data Availability

The original codes for model fitting used in the study are openly available at https://github.com/AarushShetty1/favipiravir-viral-dynamics (accessed on 29 July 2026).

## Supplementary Materials

The following supporting information can be downloaded at https://www.mdpi.com/article/10.3390/pathogens15101007/s1, File S1: Individual best-fit parameter distributions. File S2: Individual animal corner plots. File S3: Group corner plots.

## References

[1] Chen N., Zhou M., Dong X., Qu J., Gong F., Han Y., Qiu Y., Wang J., Liu Y., Wei Y. (2020). Epidemiological and clinical characteristics of 99 cases of 2019 novel coronavirus pneumonia in Wuhan, China: A descriptive study. Lancet.

[2] Wu F., Zhao S., Yu B., Chen Y.M., Wang W., Song Z.G., Hu Y., Tao Z.W., Tian J.H., Pei Y.Y. (2020). A new coronavirus associated with human respiratory disease in China. Nature.

[3] World Health Organization (2023). WHO Coronavirus (COVID-19) Dashboard.

[4] Ma W., Fu H., Jian F., Cao Y., Li M. (2023). Immune evasion and ACE2 binding affinity contribute to SARS-CoV-2 evolution. Nat. Ecol. Evol..

[5] Dong T. (2025). Evasion of T cell responses by newly emerging SARS-CoV-2 variants. Nat. Immunol..

[6] Uzer F., Erendor F., Sanlioglu S. (2026). SARS-CoV-2 Variants and Immune Evasion: Mapping the Future of Vaccine Design. Rev. Med. Virol..

[7] Davis H.E., Assaf G.S., McCorkell L., Wei H., Low R.J., Re’em Y., Redfield S., Austin J.P., Akrami A. (2021). Characterizing long COVID in an international cohort: 7 months of symptoms and their impact. EClinicalMedicine.

[8] Chai X., Qi H., Liu X., Zhou F., Jiang Y., Wu M., Lian S., Wang L., Bao Y. (2026). A narrative review of SARS-CoV-2 variants and long COVID. J. Thorac. Dis..

[9] Gozalo-Marguello M., Gonzalez-Rico C., Farinas-Alvarez C., Fernandez-Sampedro M., de Las Revillas F.A., Parra-Farinas R., Runza-Buznego P., Baldeon-Conde C., Ferrer-Pargada D., Chocarro R.P. (2026). Persistent symptoms and healthcare utilisation during five-year followup after SARS-CoV-2 infection: A multicentre cohort study in Spain. J. Infect. Public Health.

[10] Ikeokwu A.E., Lawrence R., Osieme E.D., Gidado K.M., Guy C., Dolapo O. (2023). Unveiling the Impact of COVID-19 Vaccines: A Meta-Analysis of Survival Rates Among Patients in the United States Based on Vaccination Status. Cureus J. Med. Sci..

[11] Chuenkitmongkol S., Solante R., Burhan E., Chariyalertsak S., Chiu N.C., Do-Van D., Husin M., Hwang K.P., Kiertiburanakul S., Kulkarni P.S. (2022). Expert review on global real-world vaccine effectiveness against SARS-CoV-2. Expert Rev. Vaccines.

[12] Acharya R., Kafle S., Kandinata N., Slipman B., Ghimire M., Trotter A.B. (2022). A Retrospective Cross-Sectional Study of Severe Breakthrough SARS-CoV-2 Infection in the General Population Requiring Hospitalization Within a Single Health System. J. Clin. Med. Res. Can..

[13] Lan Q., Yan Y., Zhang G., Xia S., Zhou J., Lu L., Jiang S. (2024). Clinical development of antivirals against SARS-CoV-2 and its variants. Curr. Res. Microb. Sci..

[14] Frediansyah A., Nainu F., Dhama K., Mudatsir M., Harapan H. (2021). Remdesivir and its antiviral activity against COVID-19: A systematic review. Clin. Epidemiol. Glob. Health.

[15] Durante-Mangoni E., Andini R., Bertolino L., Mele F., Florio L.L., Murino P., Corcione A., Zampino R. (2020). Early experience with remdesivir in SARS-CoV-2 pneumonia. Infection.

[16] Zhang L., Zhou R. (2020). Binding mechanism of remdesivir to SARS-CoV-2 RNA dependent RNA polymerase. Preprints.

[17] Beigel J.H., Tomashek K.M., Dodd L.E., Mehta A.K., Zingman B.S., Kalil A.C., Hohmann E., Chu H.Y., Luetkemeyer A., Kline S. (2020). Remdesivir for the Treatment of Covid-19. N. Engl. J. Med..

[18] Dobrowolska K., Zarebska-Michaluk D., Brzdek M., Rzymski P., Rogalska M., Moniuszko-Malinowska A., Kozielewicz D., Hawro M., Rorat M., Sikorska K. (2023). Retrospective Analysis of the Effectiveness of Remdesivir in COVID-19 Treatment during Periods Dominated by Delta and Omicron SARS-CoV-2 Variants in Clinical Settings. J. Clin. Med..

[19] Bartoletti M., Mozaffari E., Amin A.N., Doi Y., Loubet P., Rivera C.G., Roshon M., Rawal A., Kaiser E., Nicolae M.V. (2025). A Systematic Review and Meta-analysis of the Effectiveness of Remdesivir to Treat Severe Acute Respiratory Syndrome Coronavirus 2 in Hospitalized Patients: Have the Guidelines Evolved With the Evidence?. Clin. Infect. Dis..

[20] Loubet P., Chandak A., Spivey S., Doi Y., Amin A.N., Ahuja N., Muller V., Sax P.E. (2026). Real-world effectiveness of early remdesivir in reducing mortality among vulnerable patients hospitalized for COVID-19: Evidence for clinical pharmacists and inpatient care providers. Am. J. Health-Syst. Pharm..

[21] Bege M., Borbas A. (2024). The Design, Synthesis and Mechanism of Action of Paxlovid, a Protease Inhibitor Drug Combination for the Treatment of COVID-19. Pharmaceutics.

[22] Bai F., Beringheli T., Vitaletti V., Santoro A., Mola F., Copes A., Gemignani N., Pettenuzzo S., Castoldi R., Varisco B. (2024). Clinical Outcome and 7-Day Virological Clearance in High-Risk Patients with Mild-Moderate COVID-19 Treated with Molnupiravir, Nirmatrelvir/Ritonavir, or Remdesivir. Infect. Dis. Ther..

[23] Girardin F., Manuel O., Marzolini C., Buclin T. (2022). Evaluating the risk of drug-drug interactions with pharmacokinetic boosters: The case of ritonavir-enhanced nirmatrelvir to prevent severe COVID-19. Clin. Microbiol. Infect..

[24] Pandit J.A., Radin J.M., Chiang D.C., Spencer E.G., Pawelek J.B., Diwan M., Roumani L., Mina M.J. (2023). Differences in Participants Treated With Nirmatrelvir Plus Ritonavir Versus Untreated Controls. Clin. Infect. Dis..

[25] Edelstein G.E., Boucau J., Uddin R., Marino C., Liew M.Y., Barry M., Choudhary M.C., Gilbert R.F., Reynolds Z., Li Y. (2023). SARS-CoV-2 virologic rebound with nirmatrelvir-ritonavir therapy: An Observational Study. Ann. Intern. Med..

[26] Istifli E.S., Okumus N., Sarikurkcu C., Kuhn E.R., Netz P.A., Tepe A.S. (2024). Comparative docking and molecular dynamics studies of molnupiravir (EIDD-2801): Implications for novel mechanisms of action on influenza and SARS-CoV-2 protein targets. J. Biomol. Struct. Dyn..

[27] Patil S.M., Maruthi K.R., Bajpe S.N., Vyshali V.M., Sushmitha S., Akhila C., Ramu R. (2021). Comparative molecular docking and simulation analysis of molnupiravir and remdesivir with SARS-CoV-2 RNA dependent RNA polymerase (RdRp). Bioinformation.

[28] Jayk Bernal A., Gomes da Silva M.M., Musungaie D.B., Kovalchuk E., Gonzalez A., Delos Reyes V., Martín-Quirós A., Caraco Y., Williams-Diaz A., Brown M.L. (2022). Molnupiravir for Oral Treatment of Covid-19 in Nonhospitalized Patients. N. Engl. J. Med..

[29] Wong C.K., Lau K.T., Au I.C., Lau E.H., Poon L.L., Hung I.F., Cowling B.J., Leung G.M. (2023). Viral burden rebound in hospitalised patients with COVID-19 receiving oral antivirals in Hong Kong: A population-wide retrospective cohort study. Lancet Infect. Dis..

[30] Wang L., Berger N.A., Davis P.B., Kaelber D.C., Volkow N.D., Xu R. (2022). COVID-19 rebound after Paxlovid and Molnupiravir during January-June 2022. medRxiv.

[31] Shrestha L.B., Tedla N., Bull R.A. (2021). Broadly-Neutralizing Antibodies Against Emerging SARS-CoV-2 Variants. Front. Immunol..

[32] Focosi D., McConnell S., Casadevall A. (2022). The Omicron variant of concern: Diversification and convergent evolution in spike protein, and escape from anti-Spike monoclonal antibodies. Drug Resist. Updat..

[33] Channey S., Kale-Pradhan P., Sharma M., Lebovic D., Giuliano C., Johnson L., Bhargava A. (2026). Real world evaluation of Evusheld in prevention of COVID-19 in immunocompromised patients: An observational cohort study. Am. J. Med. Sci..

[34] Ho Y.C., Wang Y.T., Chou S.H., Liu S.Y., Chuang C., Chin W., Ho H.L., Lin Y.T. (2026). Clinical outcomes of immunocompromised patients administered tixagevimab-cilgavimab as pre-exposure prophylaxis for COVID-19: Real-world experience in Taiwan. Int. J. Infect. Dis..

[35] Marzolini C., Kuritzkes D.R., Marra F., Boyle A., Gibbons S., Flexner C., Pozniak A., Boffito M., Waters L., Burger D. (2022). Recommendations for the Management of Drug-Drug Interactions Between the COVID-19 Antiviral Nirmatrelvir/Ritonavir (Paxlovid) and Comedications. Clin. Pharmacol. Ther..

[36] Sjaarda C.P., Lau L., Simpson J.T., Fattouh R., Biondi M.J., Maguire F., Campigotto A., Feng Y., Tozer K., Wong H. (2023). Prevalence of Low-Frequency, Antiviral Resistance Variants in SARS-CoV-2 Isolates in Ontario, Canada, 2020–2023. JAMA Netw. Open.

[37] Furuta Y., Takahashi K., Fukuda Y., Kuno M., Kamiyama T., Kozaki K., Nomura N., Egawa H., Minami S., Watanabe Y. (2002). In vitro and in vivo activities of anti-influenza virus compound T-705. Antimicrob. Agents Chemother..

[38] Kaur R.J., Charan J., Dutta S., Sharma P., Bhardwaj P., Sharma P., Lugova H., Krishnapillai A., Islam S., Haque M. (2020). Favipiravir Use in COVID-19: Analysis of Suspected Adverse Drug Events Reported in the WHO Database. Infect. Dis. Resist..

[39] Costanzo M., De Giglio M.A.R., Roviello G.N. (2020). SARS-CoV-2: Recent Reports on Antiviral Therapies Based on Lopinavir/Ritonavir, Darunavir/Umifenovir, Hydroxychloroquine, Remdesivir, Favipiravir and other Drugs for the Treatment of the New Coronavirus. Curr. Med. Chem..

[40] Furuta Y., Komeno T., Nakamura T. (2017). Favipiravir (T-705), a broad spectrum inhibitor of viral RNA polymerase. Proc. Jpn. Acad. Ser. B.

[41] Huchting J., Vanderlinden E., Winkler M., Nasser H., Naesens L., Meier C. (2018). Prodrugs of the Phosphoribosylated Forms of Hydroxypyrazinecarboxamide Pseudobase T-705 and Its De-Fluoro Analogue T-1105 as Potent Influenza Virus Inhibitors. J. Med. Chem..

[42] Sissoko D., Laouenan C., Folkesson E., M’Lebing A.B., Beavogui A.H., Baize S., Camara A.M., Maes P., Shepherd S., Danel C. (2016). Experimental Treatment with Favipiravir for Ebola Virus Disease (the JIKI Trial): A Historically Controlled, Single-Arm Proof-of-Concept Trial in Guinea. PLoS Med..

[43] Oestereich L., Lüdtke A., Wurr S., Rieger T., Muñoz-Fontela C., Günther S. (2014). Successful treatment of advanced Ebola virus infection with T-705 (favipiravir) in a small animal model. Antivir. Res..

[44] Delang L., Abdelnabi R., Neyts J. (2018). Favipiravir as a potential countermeasure against neglected and emerging RNA viruses. Antivir. Res..

[45] Tate M., Illingworth C.J.R., Macgregor G., Cunningham L., Divers L., Mccartney E., Paterson L., Kelly C., Shaw A., Perkins J.S. (2025). Clinical effectiveness, safety, and viral mutagenicity of oral favipiravir for COVID-19: Results from a community-based, open-label, randomized Phase III trial. Antimicrob. Agents Chemother..

[46] Purwati, Miatmoko A., Nasronudin, Hendrianto E., Karsari D., Dinaryanti A., Ertanti N., Ihsan I.S., Purnama D.S., Asmarawati T.P. (2021). An in vitro study of dual drug combinations of anti-viral agents, antibiotics, and/or hydroxychloroquine against the SARS-CoV-2 virus isolated from hospitalized patients in Surabaya, Indonesia. PLoS ONE.

[47] Alamer A., Alrashed A.A., Alfaifi M., Alosaimi B., AlHassar F., Almutairi M., Howaidi J., Almutairi W., Mohzari Y., Sulaiman T. (2021). Effectiveness and safety of favipiravir compared to supportive care in moderately to critically ill COVID-19 patients: A retrospective study with propensity score matching sensitivity analysis. Curr. Med. Res. Opin..

[48] Tsuzuki S., Hayakawa K., Doi Y., Shinozaki T., Uemura Y., Matsunaga N., Terada M., Suzuki S., Asai Y., Yamada G. (2022). Effectiveness of Favipiravir on Nonsevere, Early-Stage COVID-19 in Japan: A Large Observational Study Using the COVID-19 Registry Japan. Infect. Dis. Ther..

[49] Zitzmann C., Kaderali L. (2018). Mathematical Analysis of Viral Replication Dynamics and Antiviral Treatment Strategies: From Basic Models to Age-Based Multi-Scale Modeling. Front. Microbiol..

[50] Perelson A.S. (2002). Modelling viral and immune system dynamics. Nat. Rev. Immunol..

[51] Dobrovolny H.M. (2020). Quantifying the effect of remdesivir in rhesus macaques infected with SARS-CoV-2. Virology.

[52] González-Parra G., Dobrovolny H.M. (2018). Modeling of fusion inhibitor treatment of RSV in African green monkeys. J. Theor. Biol..

[53] Neumann A., Lam N., Dahari H., Gretch D., Wiley T., Layden T., Perelson A. (1998). Hepatitis C viral dynamics in vivo and the 565 antiviral efficacy of interferon-*α* therapy. Science.

[54] Perelson A.S., Neumann A., Markowitz M., Leonard J., Ho D. (1996). HIV-1 dynamics in vivo: Virion clearance rate, infected cell life-span, and viral generation time. Science.

[55] Beauchemin C.A., McSharry J.J., Drusano G.L., Nguyen J.T., Went G.T., Ribeiro R.M., Perelson A.S. (2008). Modeling amantadine treatment of influenza A virus in vitro. J. Theor. Biol..

[56] Brown A.N., Bulitta J.B., McSharry J.J., Weng Q., Adams J.R., Kulawy R., Drusano G.L. (2011). Effect of Half-Life on the Pharmacodynamic Index of Zanamivir against Influenza Virus Delineated by a Mathematical Model. Antimicrob. Agents Chemother..

[57] de Mello C.P., Tao X., Kim T.H., Vicchiarelli M., Bulitta J.B., Kaushik A., Brown A.N. (2018). Clinical Regimens of Favipiravir Inhibit Zika Virus Replication in the Hollow-Fiber Infection Model. Antimicrob. Agents Chemother..

[58] Madelain V., Oestereich L., Graw F., Nguyen T.H.T., de Lamballerie X., Mentre F., Guenther S., Guedj J. (2015). Ebola virus dynamics in mice treated with favipiravir. Antivir. Res..

[59] Madelain V., Baize S., Jacquot F., Reynard S., Fizet A., Barron S., Solas C., Lacarelle B., Carbonnelle C., Mentre F. (2018). Ebola viral dynamics in nonhuman primates provides insights into virus immuno-pathogenesis and antiviral strategies. Nat. Comm..

[60] Schiffer J.T., Swan D.A., Magaret A., Corey L., Wald A., Ossig J., Ruebsamen-Schaeff H., Stoelben S., Timmler B., Zimmermann H. (2016). Mathematical modeling of herpes simplex virus-2 suppression with pritelivir predicts trial outcomes. Sci. Transl. Med..

[61] Kim K.S., Ibrahim M.M., Jia L. (2026). Mathematical approach to understanding HBV infection control and antiviral treatment efficacy in mice. Adv. Contin. Discret. Model..

[62] Melville K., Rodriguez T., Dobrovolny H.M. (2018). Investigating Different Mechanisms of Action in Combination Therapy for Influenza. Front. Pharmacol..

[63] de Mello C.P., Drusano G.L., Adams J.R., Shudt M., Kulawy R., Brown A.N. (2018). Oseltamivir-zanamivir combination therapy suppresses drug-resistant H1N1 influenza A viruses in the hollow fiber infection model (HFIM) system. Eur. J. Pharm. Sci..

[64] Koizumi Y., Ohashi H., Nakajima S., Tanaka Y., Wakita T., Perelson A.S., Iwami S., Watashi K. (2017). Quantifying antiviral activity optimizes drug combinations against hepatitis C virus infection. Proc. Natl. Acad. Sci. USA.

[65] Alexander P., Dobrovolny H.M. (2022). Treatment of Respiratory Viral Coinfections. Epidemiologia.

[66] Biswas A., Choudhuri I., Arnold E., Lyumkis D., Haldane A., Levy R.M. (2024). Kinetic coevolutionary models predict the temporal emergence of HIV-1 resistance mutations under drug selection pressure. Proc. Natl. Acad. Sci. USA.

[67] Dobrovolny H.M., Beauchemin C.A. (2017). Modelling the Emergence of Influenza Drug Resistance: The Roles of Surface Proteins, the Immune Response and Antiviral Mechanisms. PLoS ONE.

[68] Marlin R., Desjardins D., Contreras V., Lingas G., Solas C., Roques P., Naninck T., Pascal Q., Behillil S., Maisonnasse P. (2022). Antiviral efficacy of favipiravir against Zika and SARS-CoV-2 viruses in non-human primates. Nat. Commun..

[69] Baccam P., Beauchemin C., Macken C.A., Hayden F.G., Perelson A.S. (2006). Kinetics of influenza A virus infection in humans. J. Virol..

[70] Gonçalves A., Maisonnasse P., Donati F., Albert M., Behillil S., Contreras V., Naninck T., Marlin R., Solas C., Pizzorno A. (2021). Viral dynamic modeling of SARS-CoV-2 in non-human primates. PLoS Comput. Biol..

[71] Ke R., Zitzmann C., Ho D.D., Ribeiro R.M., Perelson A.S. (2021). In vivo kinetics of SARS-CoV-2 infection and its relationship with a person’s infectiousness. Proc. Natl. Acad. Sci. USA.

[72] Zhang S., Agyeman A.A., Hadjichrysanthou C., Standing J.F. (2023). SARS-CoV-2 viral dynamic modeling to inform model selection and timing and efficacy of antiviral therapy. CPT—Pharmacomet. Syst. Pharmacol..

[73] Paradis E., Pinilla L., Holder B., Abed Y., Boivin G., Beauchemin C. (2015). Impact of the H275Y and I223V Mutations in the Neuraminidase of the 2009 Pandemic Influenza Virus In Vitro and Evaluating Experimental Reproducibility. PLoS ONE.

[74] Zhao L., Zhong W. (2021). Mechanism of action of favipiravir against SARS-CoV-2: Mutagenesis or chain termination?. Innovation.

[75] Zhao J., Guo S., Yi D., Li Q., Ma L., Zhang Y., Wang J., Li X., Guo F., Lin R. (2021). A cell-based assay to discover inhibitors of SARS-CoV-2 RNA dependent RNA polymerase. Antivir. Res..

[76] Hooker K.L., Ganusov V.V. (2021). Impact of Oseltamivir Treatment on Influenza A and B Virus Dynamics in Human Volunteers. Front. Microbiol..

[77] Carsetti R., Zaffina S., Mortari E.P., Terreri S., Corrente F., Capponi C., Palomba P., Mirabella M., Cascioli S., Palange P. (2020). Different Innate and Adaptive Immune Responses to SARS-CoV-2 Infection of Asymptomatic, Mild, and Severe Cases. Front. Immunol..

[78] Hadjadj J., Yatim N., Barnabei L., Corneau A., Boussier J., Smith N., Pere H., Charbit B., Bondet V., Chenevier-Gobeaux C. (2020). Impaired type I interferon activity and inflammatory responses in severe COVID-19 patients. Science.

[79] Zhang Q., Bastard P., Liu Z., Le Pen J., Moncada-Velez M., Chen J., Ogishi M., Sabli I.K.D., Hodeib S., Korol C. (2020). Inborn errors of type I IFN immunity in patients with life-threatening COVID-19. Science.

[80] Mathew D., Giles J.R., Baxter A.E., Oldridge D.A., Greenplate A.R., Wu J.E., Alanio C., Kuri-Cervantes L., Pampena M.B., D’Andrea K. (2020). Deep immune profiling of COVID-19 patients reveals distinct immunotypes with therapeutic implications. Science.

[81] Augusto D.G., Murdolo L.D., Chatzileontiadou D.S.M., Sabatino J., Yusufali J.J., Peyser T., Butcher N.D., Kizer X., Guthrie K., Murray K. (2023). A common allele of HLA is associated with asymptomatic SARS-CoV-2 infection. Nature.

[82] Chen J., Jiang Q., Xia X., Liu K., Yu Z., Tao W., Gong W., Han J.D.J. (2020). Individual variation of the SARS-CoV-2 receptor ACE2 gene expression and regulation. Aging Cell.

[83] Vadgama N., Kreymerman A., Campbell J., Shamardina O., Brugger C., Consortium G.E.R., Deaconescu A.M., Lee R.T., Penkett C.J., Gifford C.A. (2022). SARS-CoV-2 Susceptibility and ACE2 Gene Variations Within Diverse Ethnic Backgrounds. Front. Genet..

[84] Horowitz J.E., Kosmicki J.A., Damask A., Sharma D., Roberts G.H.L., Justice A.E., Banerjee N., Coignet V.M., Yadav A., Leader J.B. (2022). Genome-wide analysis provides genetic evidence that ACE2 influences COVID-19 risk and yields risk scores associated with severe disease. Nat. Genet..

[85] Benetti E., Tita R., Spiga O., Ciolfi A., Birolo G., Bruselles A., Doddato G., Giliberti A., Marconi C., Musacchia F. (2020). ACE2 gene variants may underlie interindividual variability and susceptibility to COVID-19 in the Italian population. Eur. J. Hum. Genet..

[86] Irie K., Nakagawa A., Fujita H., Tamura R., Eto M., Ikesue H., Muroi N., Fukushima S., Tomii K., Hashida T. (2021). Population pharmacokinetics of favipiravir in patients with COVID-19. CPT—Pharmacomet. Syst. Pharmacol..

[87] Gulhan R., Eryuksel E., Oglu M.G.I., Culpan Y., Toplu A., Kocakaya D., Tigen E., Sengel B.E., Sili U., Yildizeli S.O. (2022). Pharmacokinetic characterization of favipiravir in patients with COVID-19. Br. J. Clin. Pharmacol..

[88] Suzuki M., Imai T., Sakurai A., Komoto S., Ide T., Lim C.K., Shintani A., Doi Y., Murata T. (2021). Virological and genomic analysis of SARS-CoV-2 from a favipiravir clinical trial cohort. J. Infect. Chemother..

[89] Zitzmann C., Ke R., Ribeiro R.M., Perelson A.S. (2024). How robust are estimates of key parameters in standard viral dynamic models?. PLoS Comput. Biol..

[90] Nguyen V.K., Klawonn F., Mikolajczyk R., Hernandez-Vargas E.A. (2016). Analysis of Practical Identifiability of a Viral Infection Model. PLoS ONE.

[91] Tuncer N., Martcheva M., Ciupe S.M. (2025). Structural and practical identifiability of within-host models of virus dynamics — A review. Curr. Opin. Syst. Biol..

[92] Liyanage Y.R., Heitzman-Breen N., Tuncer N., Ciupe S.M. (2024). Identifiability investigation of within-host models of acute virus infection. Math. Biosci. Eng..

[93] Heitzman-Breen N., Liyanage Y.R., Duggal N., Tuncer N., Ciupe S.M. (2024). The effect of model structure and data availability on Usutu virus dynamics at three biological scales. R. Soc. Open Sci..

[94] Vasquez D.M., Chiem K., Silvas J., Park J.G., Ye C., Martinez-Sobrido L. (2021). Live Imaging and Quantification of Viral Infection in K18 hACE2 Transgenic Mice using Reporter-Expressing Recombinant SARS-CoV-2. JOVE-J. Vis. Exp..

[95] Chiem K., Park J.G., Vasquez D.M., Plemper R.K., Torrelles J.B., Kobie J.J., Walter M.R., Ye C., Martinez-Sobrido L. (2022). Monitoring SARS-CoV-2 Infection Using a Double Reporter-Expressing Virus. Microbiol. Spectr..

[96] Petrie S.M., Guarnaccia T., Laurie K.L., Hurt A.C., McVernon J., McCaw J.M. (2013). Reducing Uncertainty in Within-Host Parameter Estimates of Influenza Infection by Measuring Both Infectious and Total Viral Load. PLoS ONE.

[97] Rowell C.E., Dobrovolny H.M. (2020). Energy Requirements for Loss of Viral Infectivity. Food Env. Virol..

[98] Beauchemin C.A., Kim Y.I., Yu Q., Ciaramella G., DeVincenzo J.P. (2019). Uncovering critical properties of the human respiratory syncytial virus by combining in vitro assays and in silico analyses. PLoS ONE.

[99] Pinilla L.T., Holder B.P., Abed Y., Boivin G., Beauchemin C.A.A. (2012). The H275Y Neuraminidase Mutation of the Pandemic A/H1N1 Influenza Virus Lengthens the Eclipse Phase and Reduces Viral Output of Infected Cells, Potentially Compromising Fitness in Ferrets. J. Virol..

[100] Putter H., Heisterkamp S., Lange J., de Wolf F. (2002). A Bayesian approach to parameter estimation in HIV dynamical models. Stat. Med..

[101] Morris D.J., Kennedy L., Black A.J. (2025). Random time-shift approximation enables hierarchical Bayesian inference of mechanistic within-host viral dynamics models on large datasets. PLoS Comput. Biol..

[102] Korula P., Alexander H., John J.S., Kirubakaran R., Singh B., Tharyan P., Rupali P. (2024). Favipiravir for treating COVID-19. Cochrane Database Syst. Rev..

[103] Solaymani-Dodaran M., Ghanei M., Bagheri M., Qazvini A., Vahedi E., Saadat S.H., Setarehdan S.A., Ansarifar A., Biganeh H., Mohazzab A. (2021). Safety and efficacy of Favipiravir in moderate to severe SARS-CoV-2 pneumonia. Int. Immunopharmacol..

[104] Tigen E.T., Mikulska M., Sengel B.E., Signori A., Dettori S., Tutino S., Guner A.E., Odabasi Z., Korten V., Bassetti M. (2024). Efficacy of favipiravir in COVID-19: A retrospective two center comparative study. J. Infect. Dev. Ctries..

[105] Luvira V., Schilling W.H.K., Jittamala P., Watson J.A., Boyd S., Siripoon T., Ngamprasertchai T., Almeida P.J., Ekkapongpisit M., Cruz C. (2024). Clinical antiviral efficacy of favipiravir in early COVID-19 (PLATCOV): An open-label, randomised, controlled, adaptive platform trial. BMC Infect. Dis..

[106] Al Sulaiman K., Aljuhani O., Korayem G.B., Altebainawi A.F., Alfaifi M., Nahari M., Almagthali A., Thabit A.K., Alhajaji R., Alharbi R. (2023). When antivirals backfire: An evaluation of favipiravir’s clinical outcomes in critically ill patients with COVID-19: A multicenter cohort study. J. Infect. Public Health.

[107] Shah P.L., Orton C.M., Grinsztejn B., Donaldson G.C., Ramirez B.C., Tonkin J., Santos B.R., Cardoso S.W., Ritchie I.A., Conway F. (2023). Favipiravir in patients hospitalised with COVID-19 (PIONEER trial): A multicentre, open-label, phase 3, randomised controlled trial of early intervention versus standard care. Lancet Respir. Med..

[108] Naveed A., Cheema H.A., Shahid A., Umer M., Ul Hussain H., Rehman M.E.U., Singh H., Kurman J.S., Sahra S., Ahmad F. (2024). Favipiravir for the Treatment of Hospitalized Patients with COVID-19: A Systematic Review and Meta-Analysis of Randomized Controlled Trials. Am. J. Ther..

[109] Bosaeed M., Alharbi A., Mahmoud E., Alrehily S., Bahlaq M., Gaifer Z., Alturkistani H., Alhagan K., Alshahrani S., Tolbah A. (2022). Efficacy of favipiravir in adults with mild COVID-19: A randomized, double-blind, multicentre, placebo-controlled clinical trial. Clin. Microbiol. Infect..

[110] Fu D., Cao R., Zhao L., Li W., Zhong W., Wen J. (2020). Oral favipiravir for patients with delayed SARS-CoV-2 viral RNA clearance: A case series. Crit. Care.

[111] Hobbs F.D.R., Gbinigie-Thompson O.A., Shanyinde M., Yu L.M., Harris V., Dorward J., Hayward G., Saville B.R., Berry N.S., Evans P.H. (2024). Favipiravir for COVID-19 in adults in the community in PRINCIPLE, an open-label, randomised, controlled, adaptive platform trial of short- and longer-term outcomes. J. Infect..

[112] Ibrahim S., Siemieniuk R.A.C., Oliveros M.J., Islam N., Martinez J.P.D., Izcovich A., Qasim A., Zhao Y., Zaror C., Yao L. (2025). Drug treatments for mild or moderate covid-19: Systematic review and network meta-analysis. BMJ Br. Med. J..

[113] Udwadia Z.F., Singh P., Barkate H., Patil S., Rangwala S., Pendse A., Kadam J., Wu W., Caracta C.F., Tandon M. (2021). Efficacy and safety of favipiravir, an oral RNA-dependent RNA polymerase inhibitor, in mild-to-moderate COVID-19: A randomized, comparative, open-label, multicenter, phase 3 clinical trial. Int. J. Infect. Dis..

[114] Shinkai M., Tsushima K., Tanaka S., Hagiwara E., Tarumoto N., Kawada I., Hirai Y., Fujiwara S., Komase Y., Saraya T. (2021). Efficacy and Safety of Favipiravir in Moderate COVID-19 Pneumonia Patients without Oxygen Therapy: A Randomized, Phase III Clinical Trial. Infect. Dis. Ther..

[115] Siripongboonsitti T., Muadchimkaew M., Tawinprai K., Issaranon O., Meepholkij W., Arttawejkul P., Vararungzarit A., Dhissayakamol O., Preeyachit W., Soonklang K. (2024). Assessing favipiravir impact on SARS-CoV-2 transmission within households: Insights from a multi-center study (FaviPrev). J. Virus Erad..

[116] Elimam H., El-Sawy H.S., Fayed M.A.A., Mahmoud S.H., Bakr R.O., Saleh R.M., Mostafa A., Elshal M.F. (2024). Antiviral potential of rosuvastatin and hesperidin in combination with favipiravir liposomal nanoformulations in targeting the main protease (Mpro) of SARS-CoV-2: Molecular docking, molecular dynamics and in-vitro studies. J. Drug Deliv. Sci. Technol..

[117] Abdel-Halim H., Hajar M., Hasouneh L., Abdelmalek S.M.A. (2022). Identification of Drug Combination Therapies for SARS-CoV-2: A Molecular Dynamics Simulations Approach. Drug Des. Dev. Ther..

[118] Gallardo-Toledo E., Neary M., Sharp J., Herriott J., Kijak E., Bramwell C., Curley P., Arshad U., Pertinez H., Rajoli R.K.R. (2023). Chemoprophylactic Assessment of Combined Intranasal SARS-CoV-2 Polymerase and Exonuclease Inhibition in Syrian Golden Hamsters. Viruses.

[119] Agyeman A.A., Buggiotti L., Brown L.A.K., Guerra-Assuncao J.A., Kembou-Ringert J.E., Mak W.Y., Breuer J., Lowe D.M., Standing J.F., Investigators F. (2026). Population pharmacokinetic/pharmacodynamic modelling to evaluate favipiravir in combination with lopinavir-ritonavir in patients with COVID-19. Br. J. Clin. Pharmacol..

[120] Chakraborty C., Bhattacharya M., Saha A., Alshammari A., Alharbi M., Saikumar G., Pal S., Dhama K., Lee S.S. (2023). Revealing the structural and molecular interaction landscape of the favipiravir-RTP and SARS-CoV-2 RdRp complex through integrative bioinformatics: Insights for developing potent drugs targeting SARS-CoV-2 and other viruses. J. Infect. Public Health.

[121] Boutin M., Lebreton J., da Silva-Junior E.F., Pizzorno A., Traversier A., Rosa-Calatrava M., Tessier A. (2026). Synthesis and first evaluation of new favipiravir analogs based on cyclic amidrazones scaffolds. Eur. J. Med. Chem. Rep..

[122] Al-Shuaeeb R.A.A., Abd El-Mageed H.R., Ahmed S.A., Mohamed H.S., Hamza Z.S., Rafi M.O., Rahman M.S. (2023). Identification of potent COVID-19 main protease inhibitors by loading of favipiravir on Mg12O12 and Zn12O12 nanoclusters: An in silico strategy for COVID-19 treatment. J. Biomol. Struct. Dyn..

[123] Ataei S., Nemati-Kande E., Bahrami A. (2023). Quantum DFT studies on the drug delivery of favipiravir using pristine and functionalized chitosan nanoparticles. Sci. Rep..

[124] Tulbah A.S., Lee W.H. (2021). Physicochemical Characteristics and In Vitro Toxicity/Anti-SARS-CoV-2 Activity of Favipiravir Solid Lipid Nanoparticles (SLNs). Pharmaceuticals.

[125] Winnett A.V., Akana R., Shelby N., Davich H., Caldera S., Yamada T., Reyna J.R.B., Romano A.E., Carter A.M., Kim M.K. (2023). Extreme differences in SARS-CoV-2 viral loads among respiratory specimen types during presumed pre-infectious and infectious periods. PNAS Nexus.

[126] Gadiyar I., Dobrovolny H.M. (2024). Different routes of infection of H5N1 lead to changes in infecting time. Math. Biosci..

[127] Urano E., Okamura T., Ono C., Ueno S., Nagata S., Kamada H., Higuchi M., Furukawa M., Kamitani W., Matsuura Y. (2021). COVID-19 cynomolgus macaque model reflecting human COVID-19 pathological conditions. Proc. Natl. Acad. Sci. USA.

[128] Cao W.J., Wang F.S., Song J.W. (2023). Characteristics and Potential Roles of Natural Killer Cells During SARS-CoV-2 Infection. Infect. Dis. Immun..

[129] Liu Y., Arase H. (2022). Neutralizing and enhancing antibodies against SARS-CoV-2. Inflamm. Regen..

[130] Holder B.P., Beauchemin C.A. (2011). Exploring the effect of biological delays in kinetic models of influenza within a host or cell culture. BMC Public Health.

[131] Kakizoe Y., Nakaoka S., Beauchemin C.A., Morita S., Mori H., Igarashi T., Aihara K., Miura T., Iwami S. (2015). A method to determine the duration of the eclipse phase for in vitro infection with a highly pathogenic SHIV strain. Sci. Rep..

[132] Beauchemin C.A., Miura T., Iwami S. (2017). Duration of SHIV production by infected cells is not exponentially distributed: Implications for estimates of infection parameters and antiviral efficacy. Sci. Rep..

[133] Timm A., Yin J. (2012). Kinetics of virus production from single cells. Virology.

[134] Matthews P., Doty M., Dobrovolny H.M. (2021). Modeling the dynamics of viral-host interaction during treatment of productively infected cells and free virus involving total immune response. Nonlinear Anal. Model. Control.

[135] Pertinez H., Rajoli R.K.R., Khoo S.H., Owen A. (2021). Pharmacokinetic modelling to estimate intracellular favipiravir ribofuranosyl-5’-triphosphate exposure to support posology for SARS-CoV-2. J. Antimicrob. Chemother..

[136] Palmer J., Dobrovolny H.M., Beauchemin C.A. (2017). The in vivo efficacy of neuraminidase inhibitors cannot be determined from the decay rates of influenza viral titers observed in treated patients. Sci. Rep..

