# Supplementary material for: Estimation of SARS-CoV-2 Viral Dynamics Parameters in Favipiravir-Treated Cynomolgus Macaques: Comparison of Individual and Group Average Model Fitting
Source: Pathogens. 2026 Sep 23;15(10):1007. doi: 10.3390/pathogens15101007 (PMC13708454; doi:10.3390/pathogens15101007)
Supplement: Supplementary file 1 [file pathogens-15-01007-s001.zip › pathogens-4498738-supplementary.pdf]

Supplementary Material: Estimation of SARS-CoV-2 Viral Dynamics Parameters in Favipiravir-Treated Cynomolgus Macaques

Aarush Shetty and Hana M. Dobrovolny

September 16, 2026

S1 Individual best-fit parameter distributions

The distributions of best-fit parameter estimates across all individual animals, grouped by treatment, are shown in Figure S1. Each violin displays the within-group distribution of each parameter on a $\log_{10}$ scale, with individual animal estimates overlaid as scatter points. All five parameters show broadly overlapping distributions across groups, with substantial spread within each group.

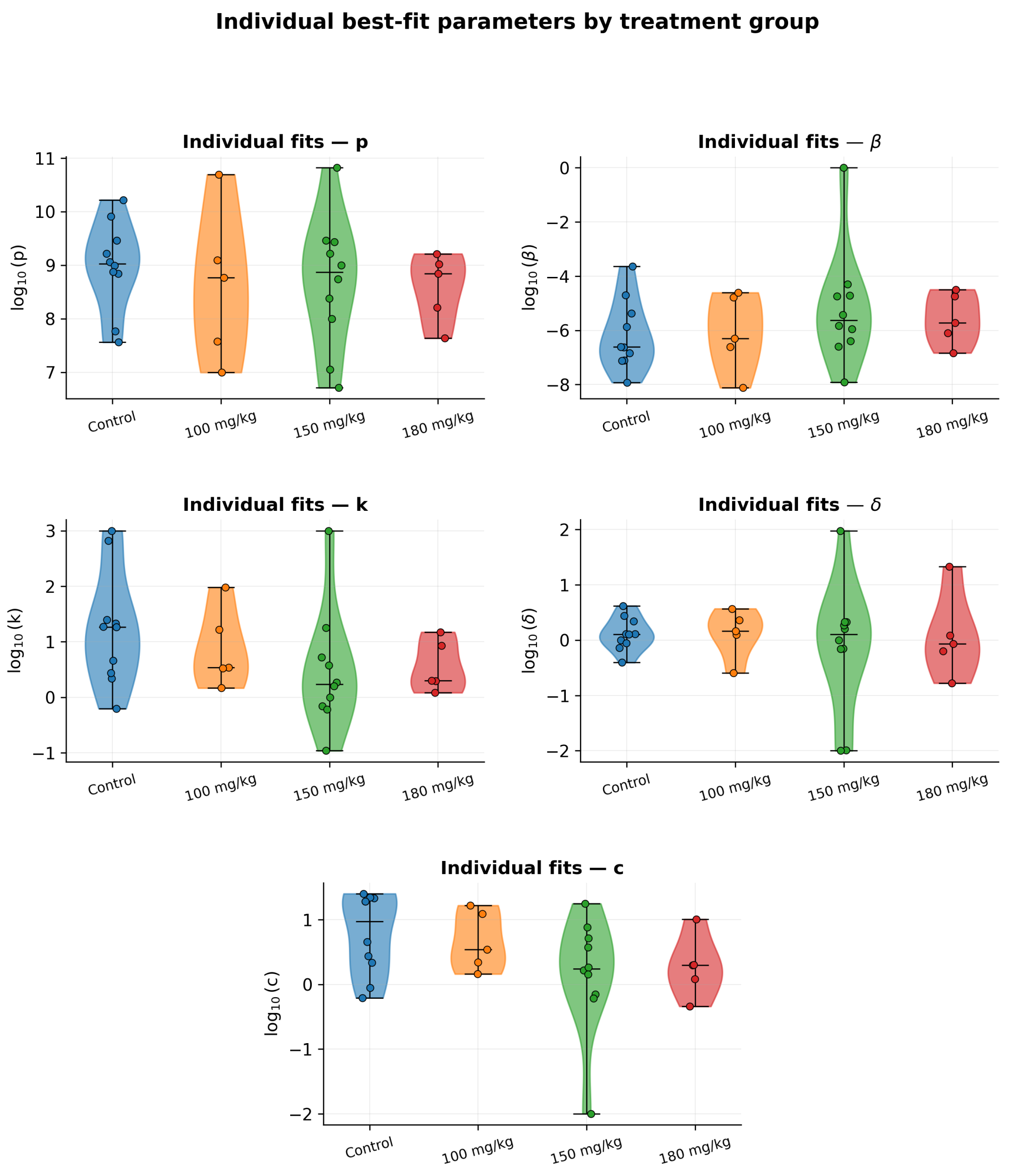

Figure S1: Distributions of individual best-fit parameter estimates by treatment group ($\log_{10}$ scale). Each violin represents the within-group distribution; scatter points show individual animal estimates. Horizontal bars indicate group medians.

S2 Individual Animal Corner Plots

Corner plots showing pairwise bootstrap parameter distributions for all 30 individual animals and the four treatment groups are presented below. Each panel displays the marginal distribution (histogram with kernel density estimate) on the diagonal and a filled 2-D contour plot for each pair of parameters on the lower triangle. Green dashed lines indicate the maximum-likelihood (best-fit) parameter values. All parameters are shown on a $\log_{10}$ scale. Parameters: β = infection rate (mL copies^{-1} d^{-1}), k = eclipse transition rate (d^{-1}), δ = infected-cell loss rate (d^{-1}), p = viral production rate (copies cell^{-1} d^{-1}), c = viral clearance rate (d^{-1}).

Control group

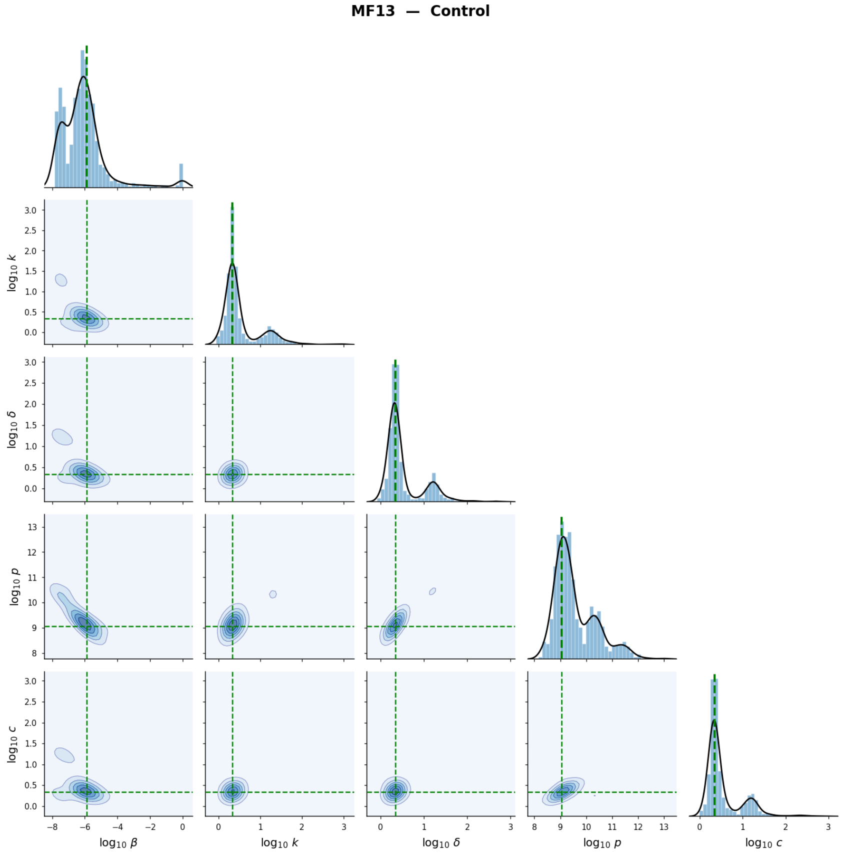

Figure S2: Bootstrap parameter distributions for animal MF13 (Control).

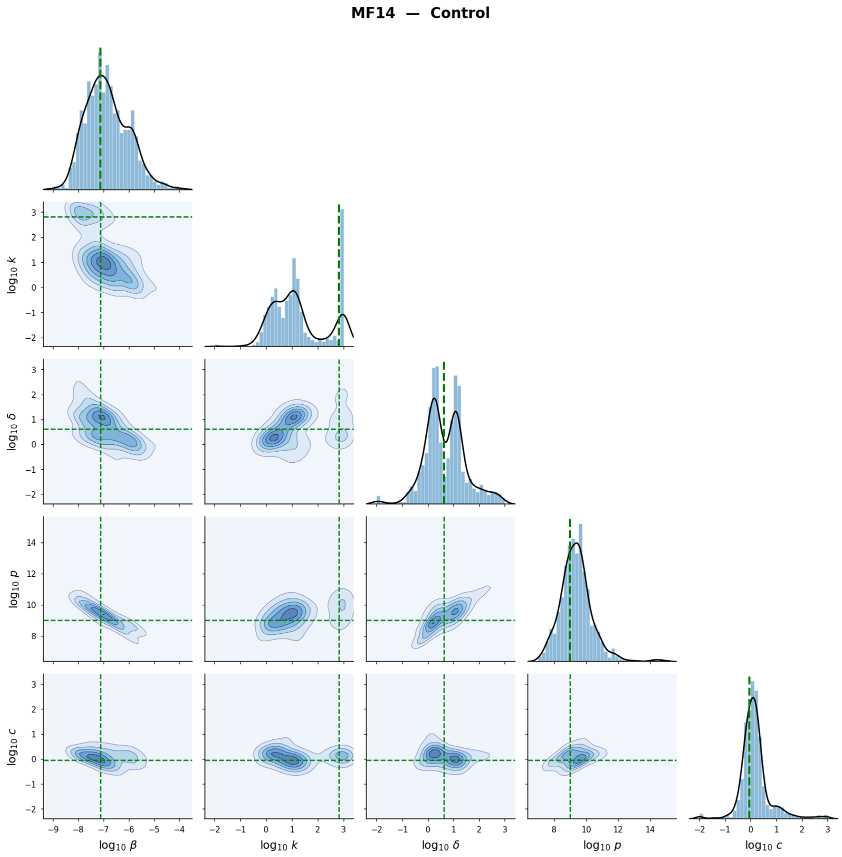

Figure S3: Bootstrap parameter distributions for animal MF14 (Control).

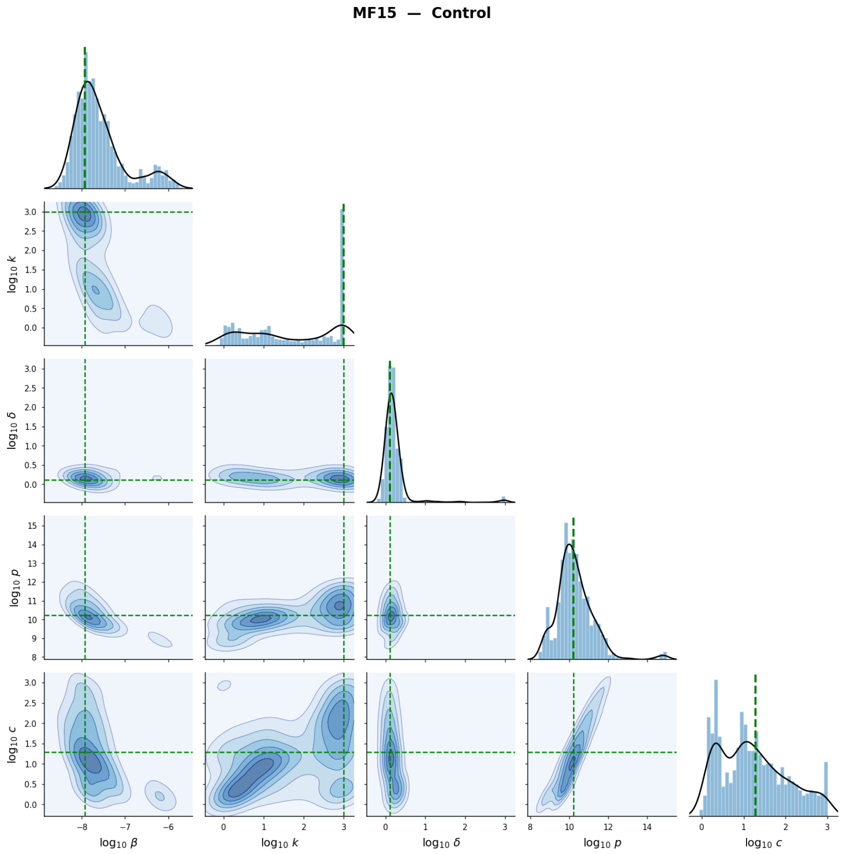

Figure S4: Bootstrap parameter distributions for animal MF15 (Control).

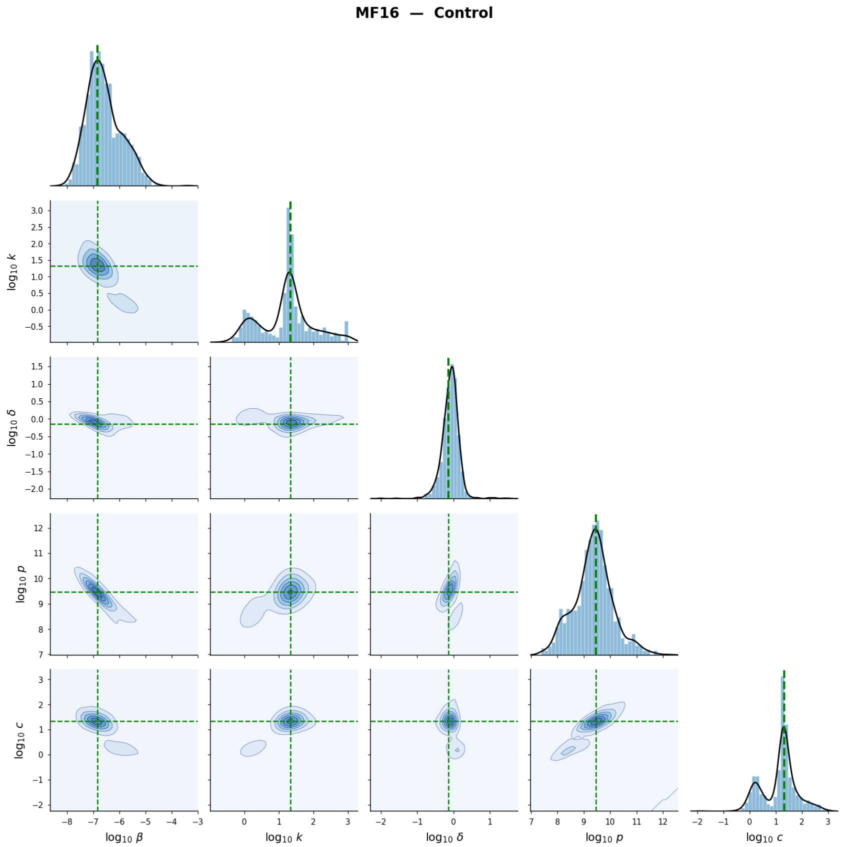

Figure S5: Bootstrap parameter distributions for animal MF16 (Control).

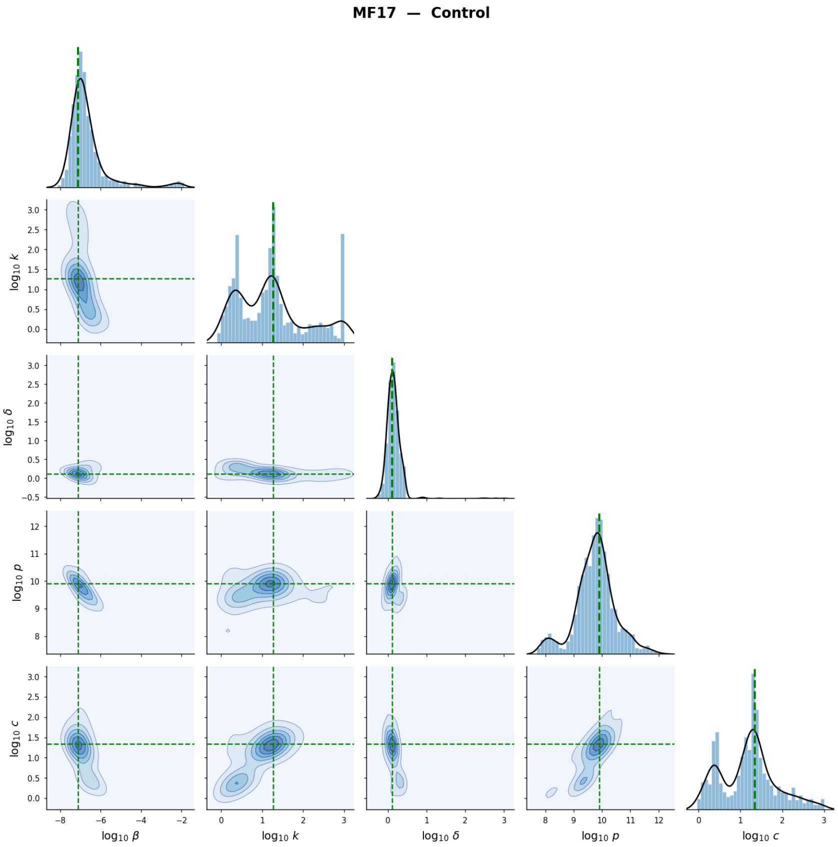

Figure S6: Bootstrap parameter distributions for animal MF17 (Control).

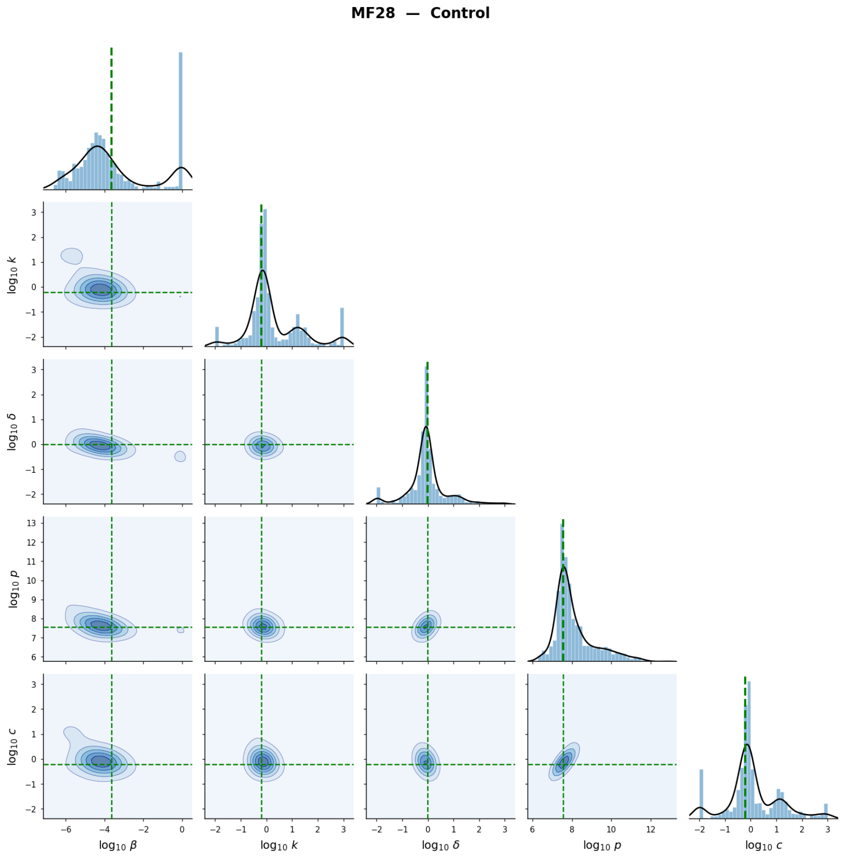

Figure S7: Bootstrap parameter distributions for animal MF28 (Control).

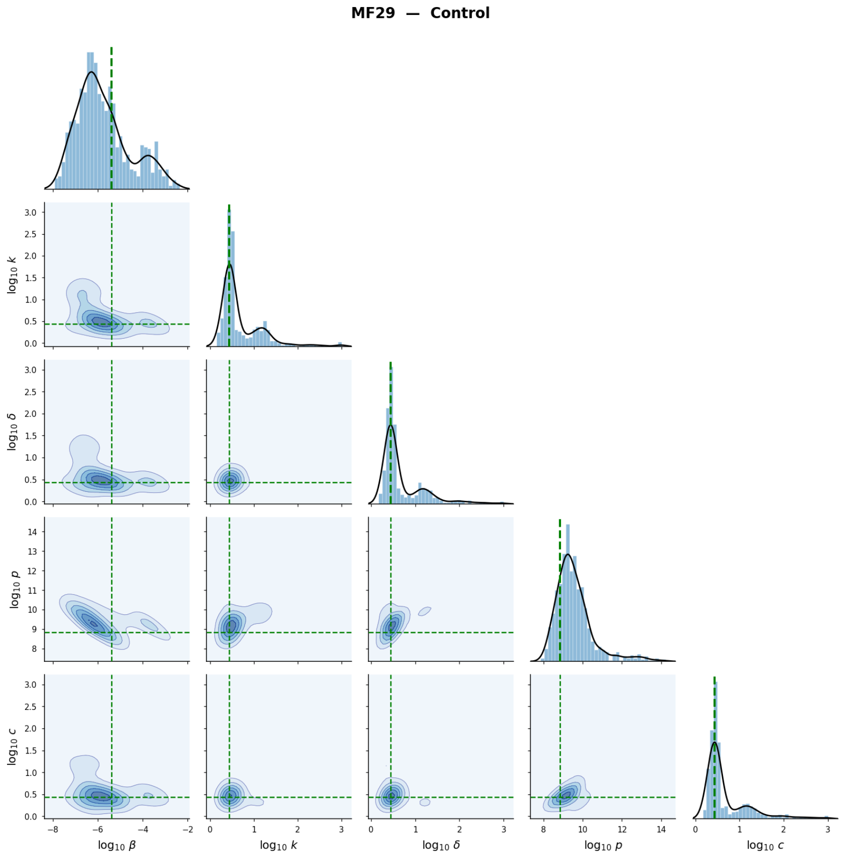

Figure S8: Bootstrap parameter distributions for animal MF29 (Control).

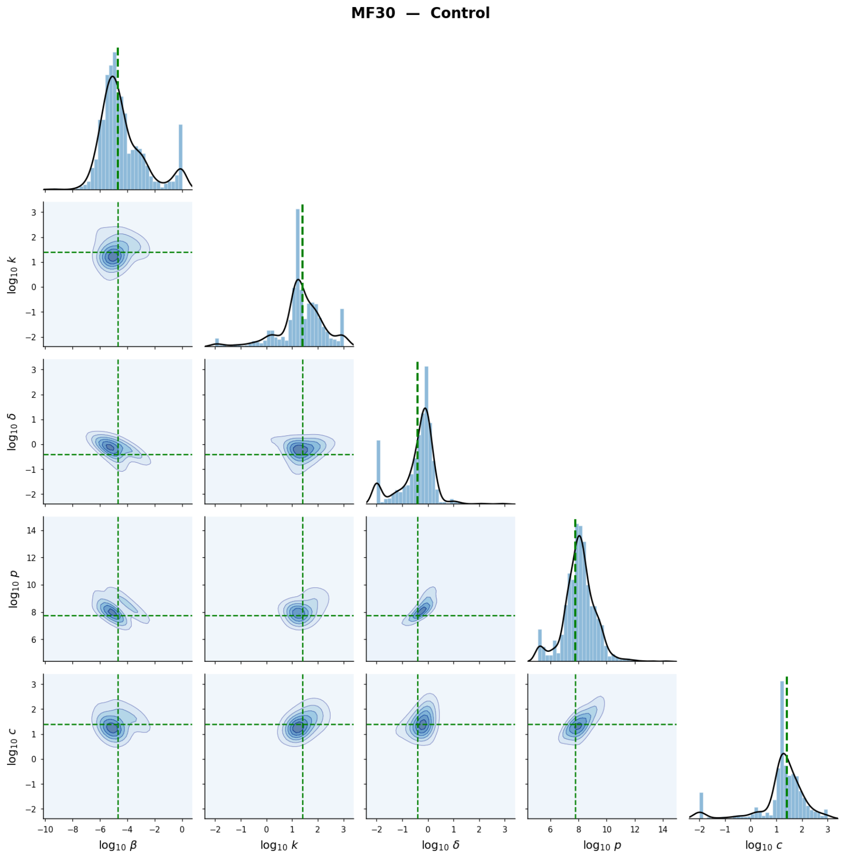

Figure S9: Bootstrap parameter distributions for animal MF30 (Control).

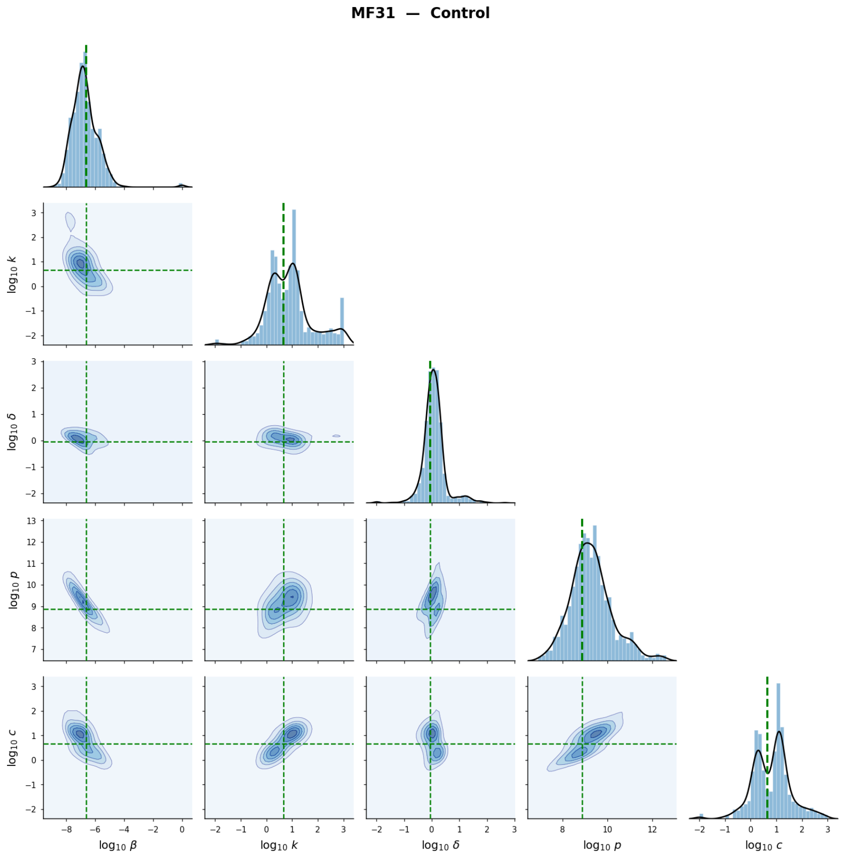

Figure S10: Bootstrap parameter distributions for animal MF31 (Control).

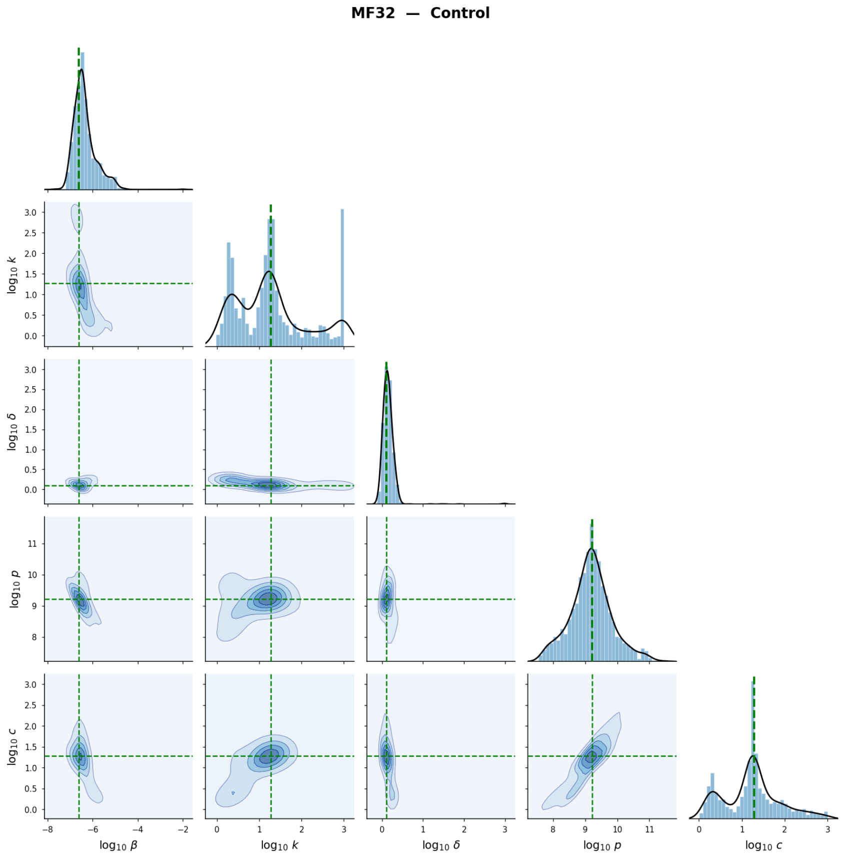

Figure S11: Bootstrap parameter distributions for animal MF32 (Control).

Favipiravir 100 mg/kg group

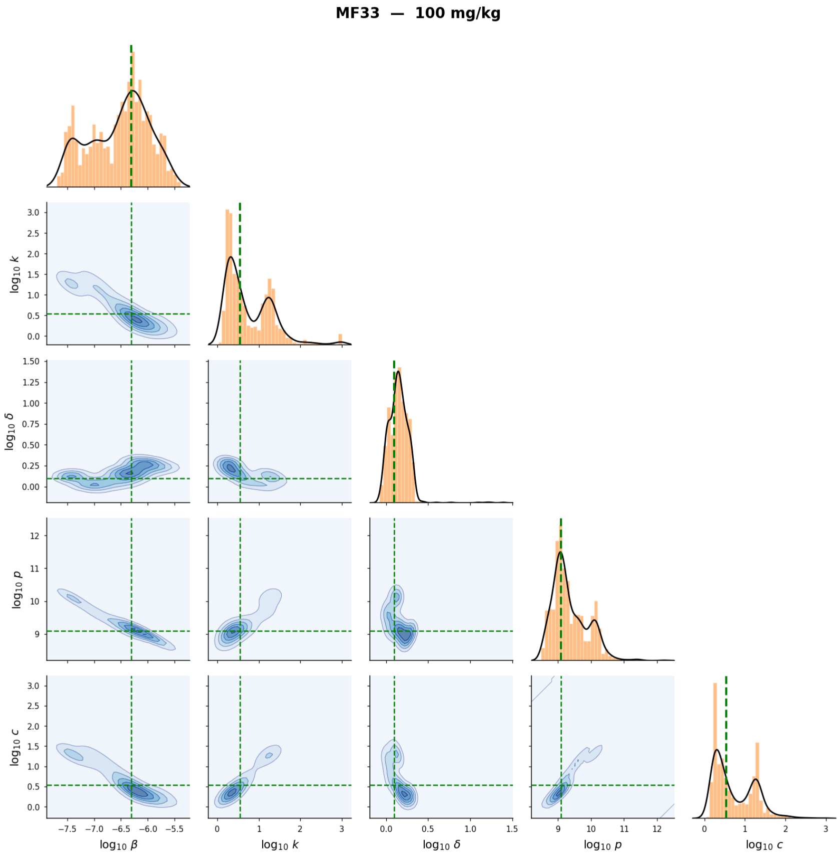

Figure S12: Bootstrap parameter distributions for animal MF33 (100 mg/kg).

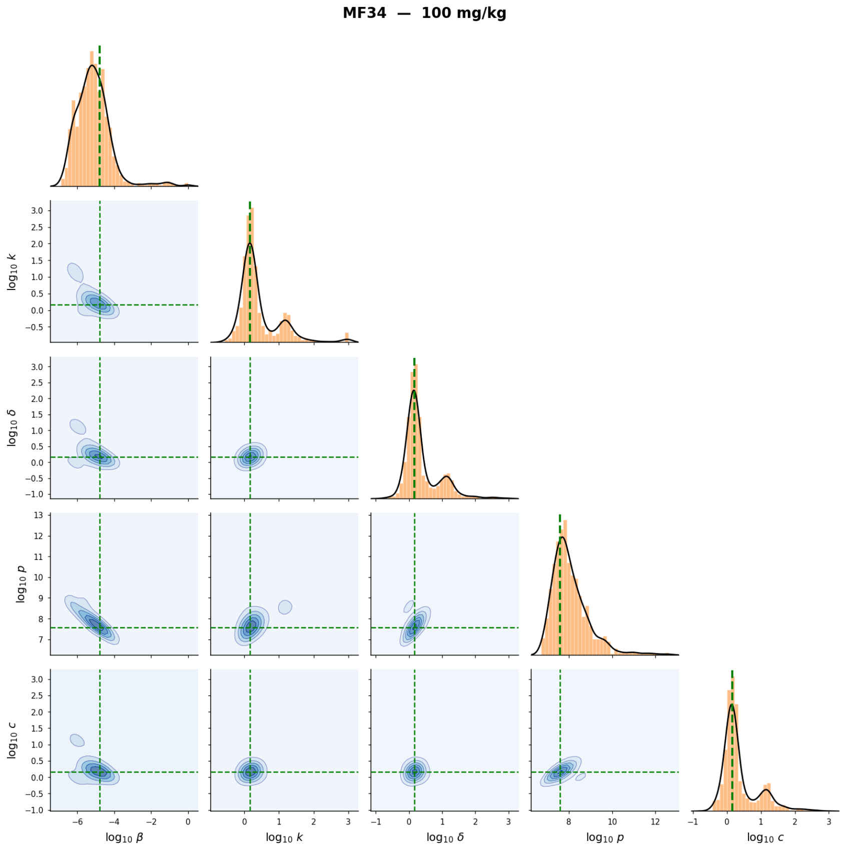

Figure S13: Bootstrap parameter distributions for animal MF34 (100 mg/kg).

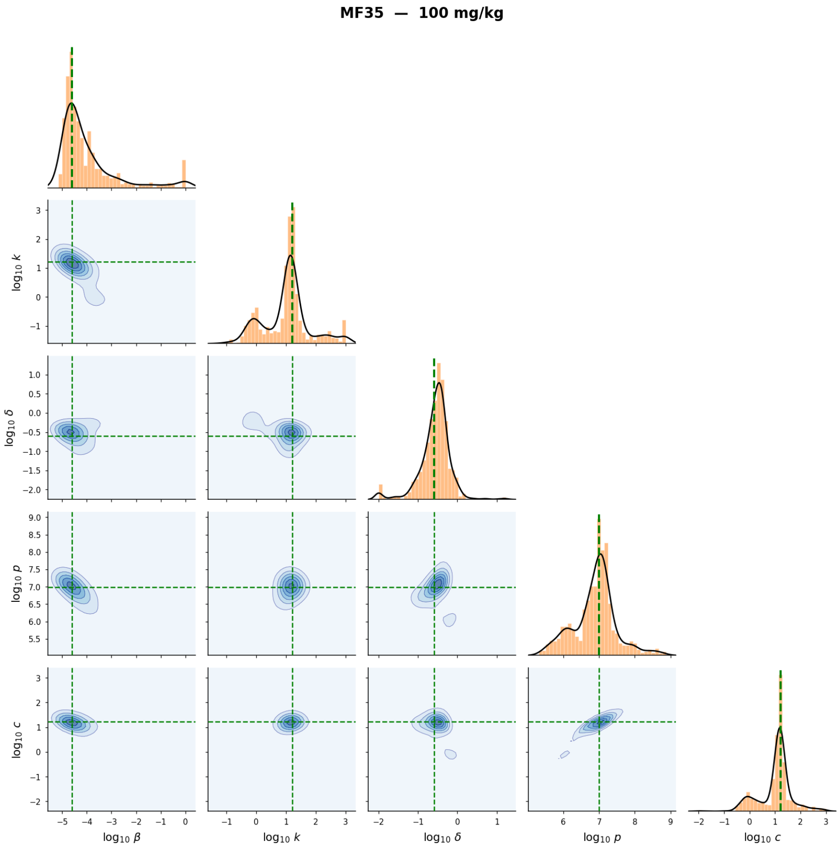

Figure S14: Bootstrap parameter distributions for animal MF35 (100 mg/kg).

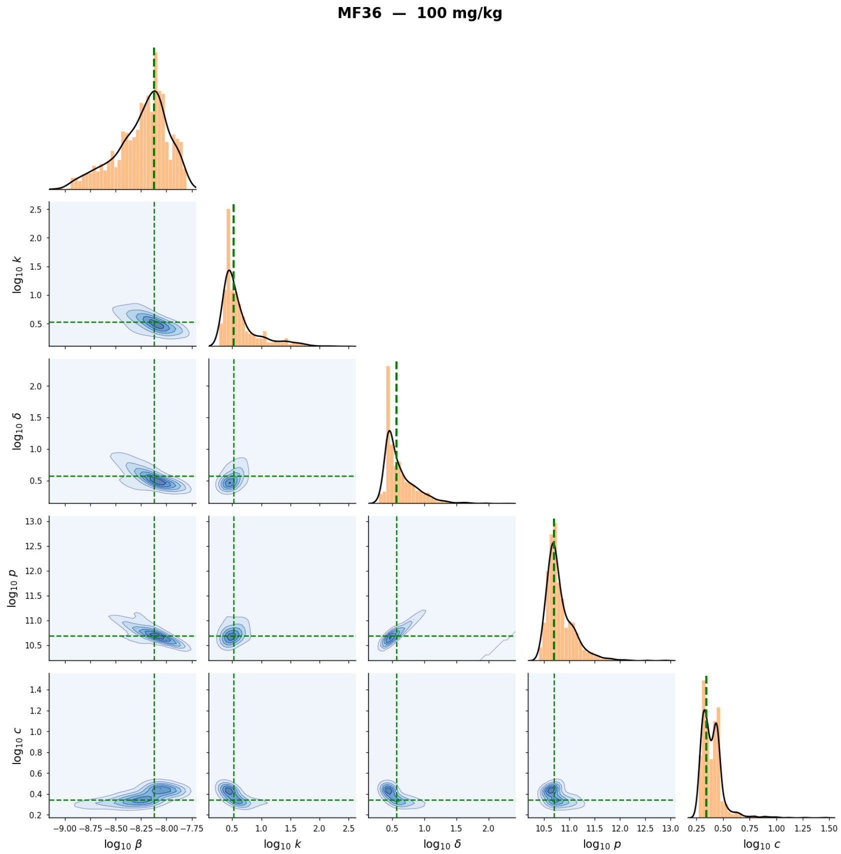

Figure S15: Bootstrap parameter distributions for animal MF36 (100 mg/kg).

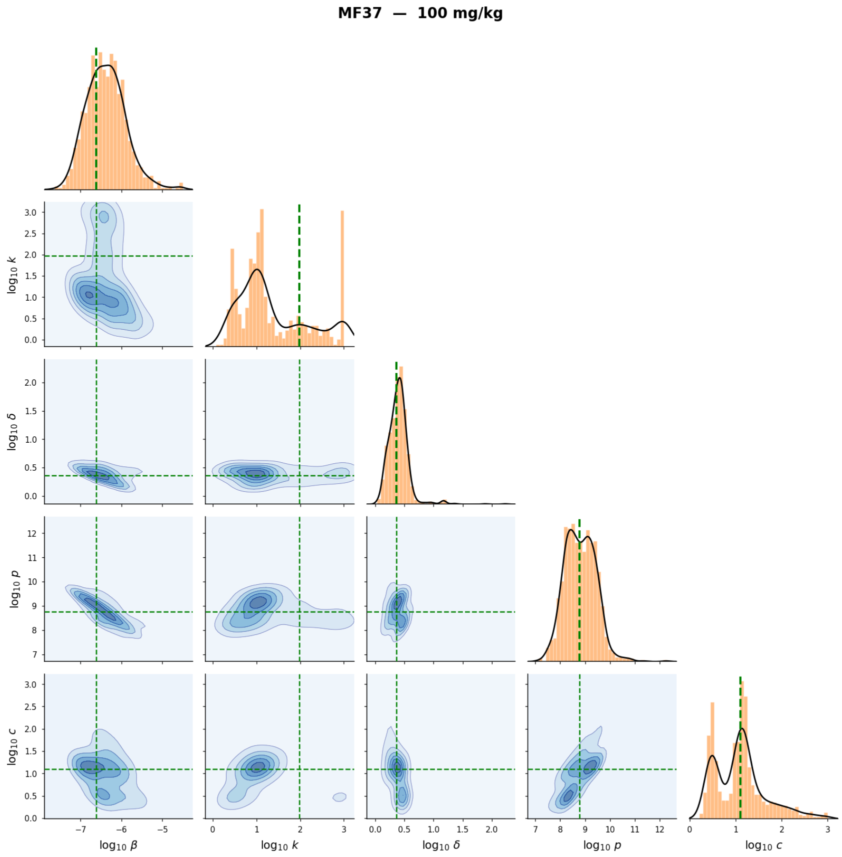

Figure S16: Bootstrap parameter distributions for animal MF37 (100 mg/kg).

Favipiravir 150 mg/kg group

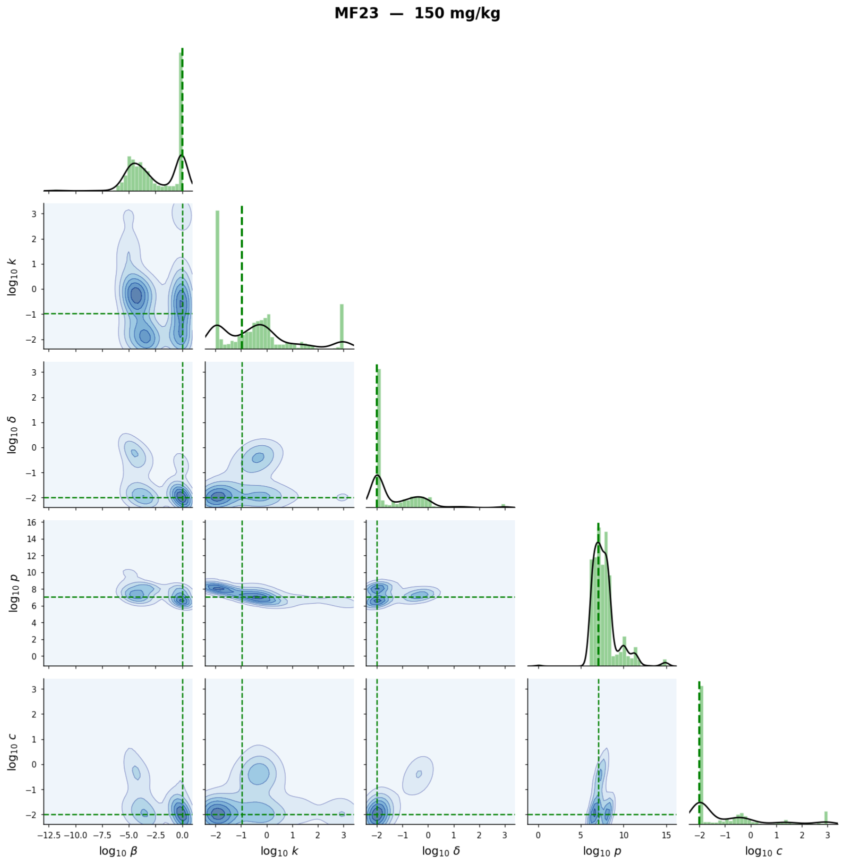

Figure S17: Bootstrap parameter distributions for animal MF23 (150 mg/kg).

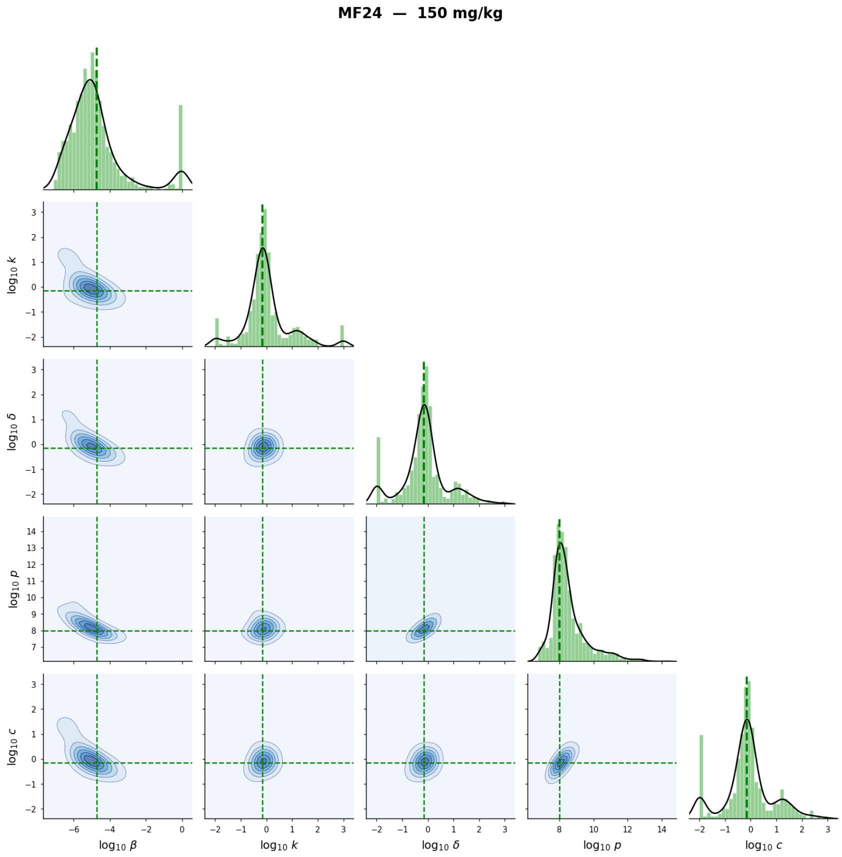

Figure S18: Bootstrap parameter distributions for animal MF24 (150 mg/kg).

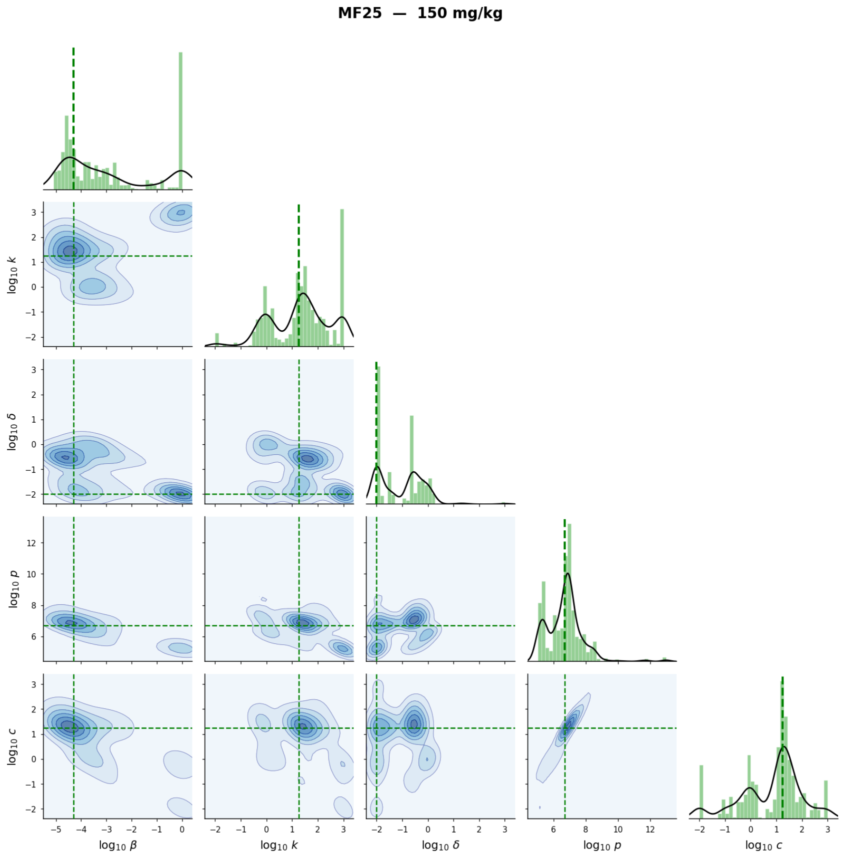

Figure S19: Bootstrap parameter distributions for animal MF25 (150 mg/kg).

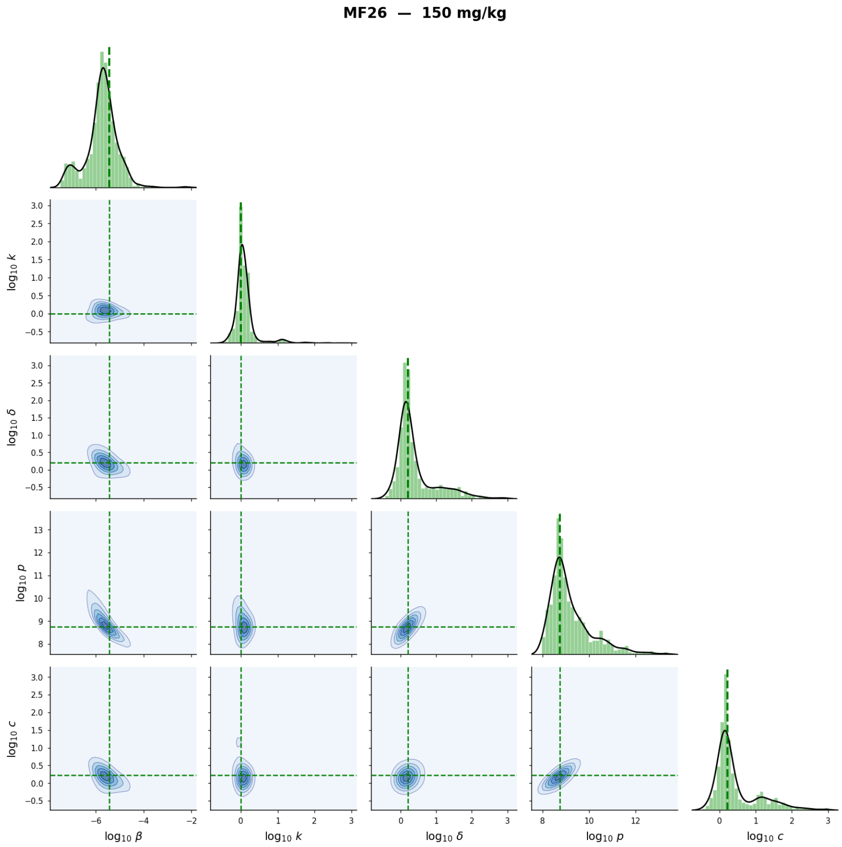

Figure S20: Bootstrap parameter distributions for animal MF26 (150 mg/kg).

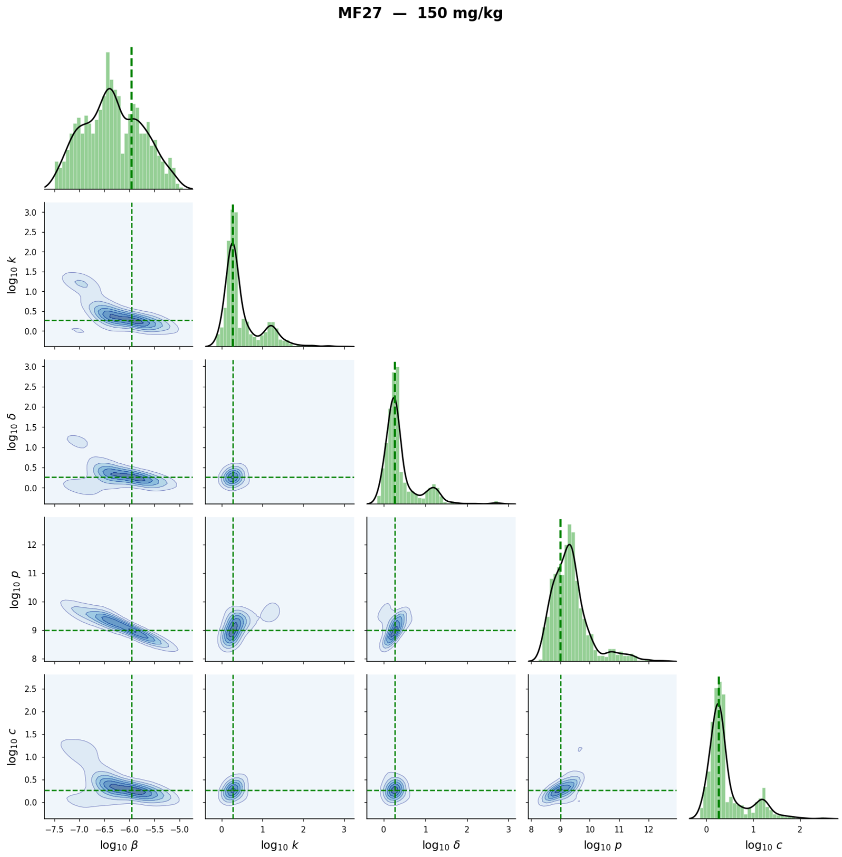

Figure S21: Bootstrap parameter distributions for animal MF27 (150 mg/kg).

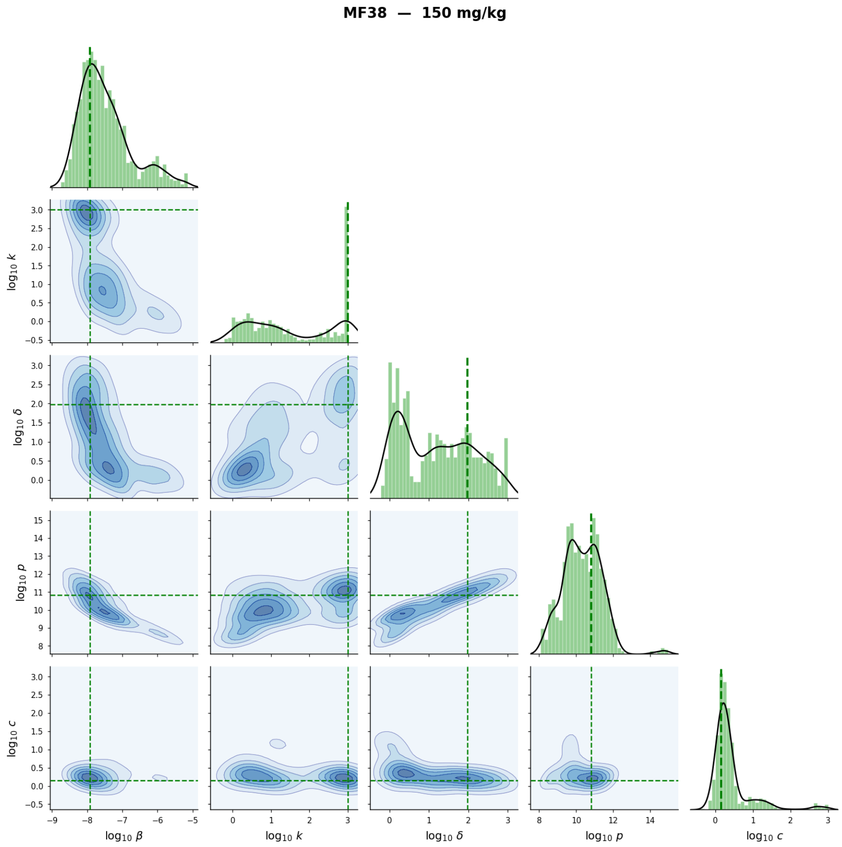

Figure S22: Bootstrap parameter distributions for animal MF38 (150 mg/kg).

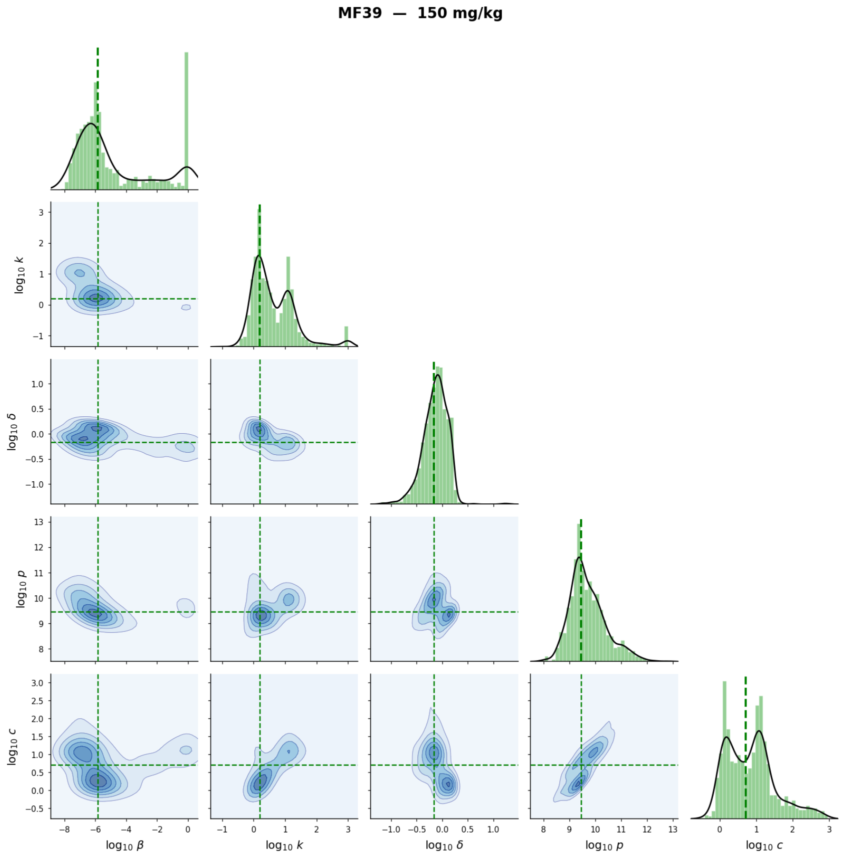

Figure S23: Bootstrap parameter distributions for animal MF39 (150 mg/kg).

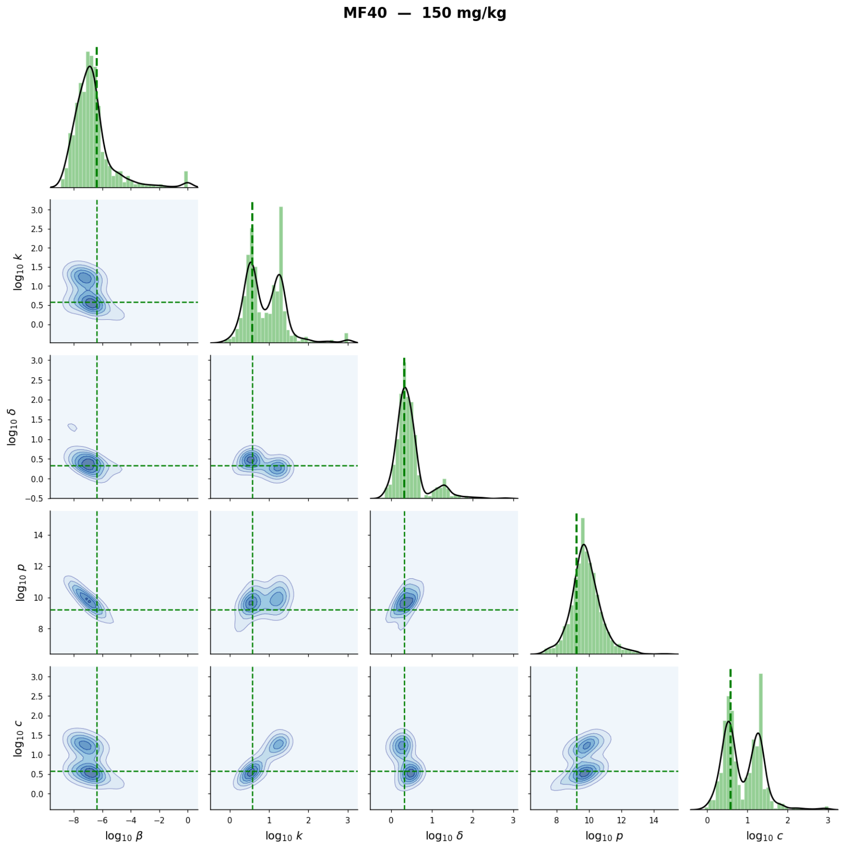

Figure S24: Bootstrap parameter distributions for animal MF40 (150 mg/kg).

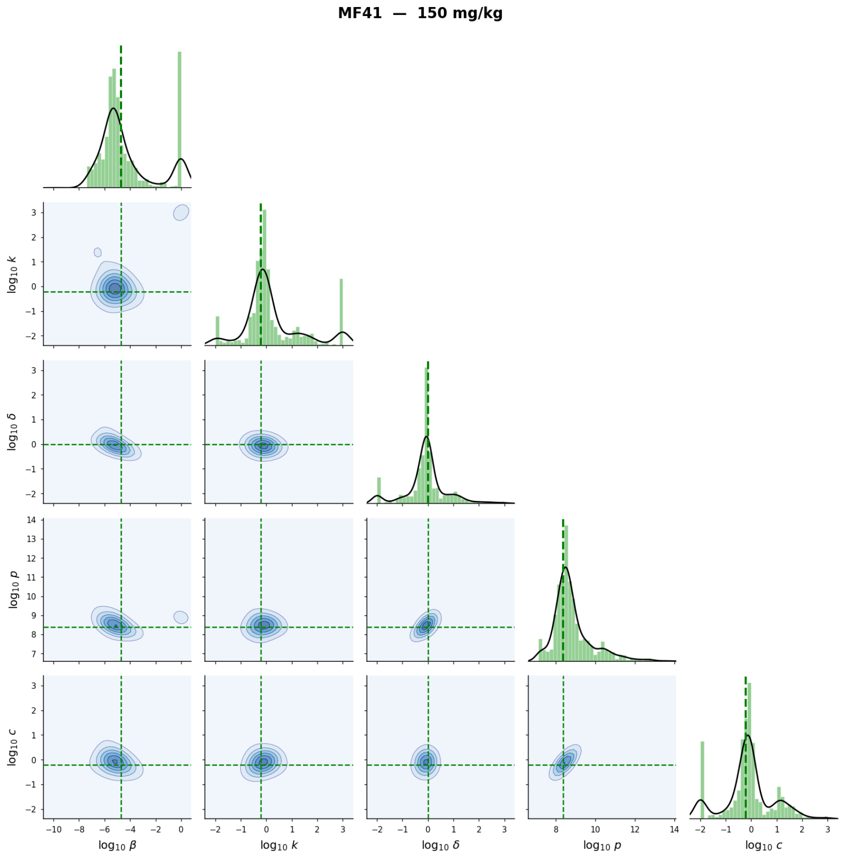

Figure S25: Bootstrap parameter distributions for animal MF41 (150 mg/kg).

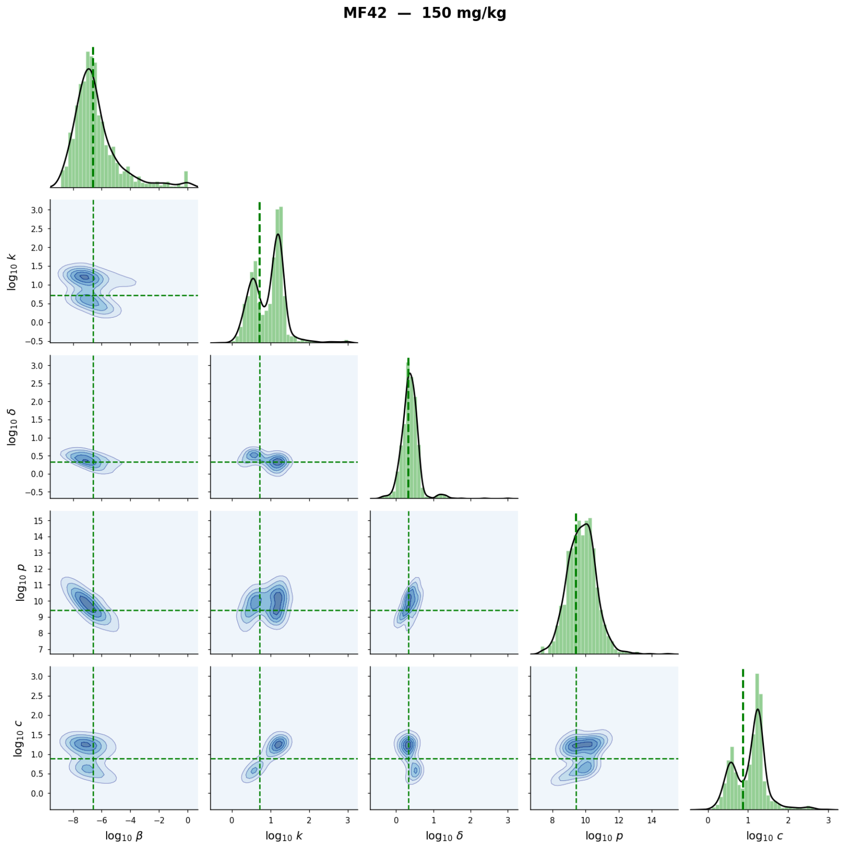

Figure S26: Bootstrap parameter distributions for animal MF42 (150 mg/kg).

Favipiravir 180 mg/kg group

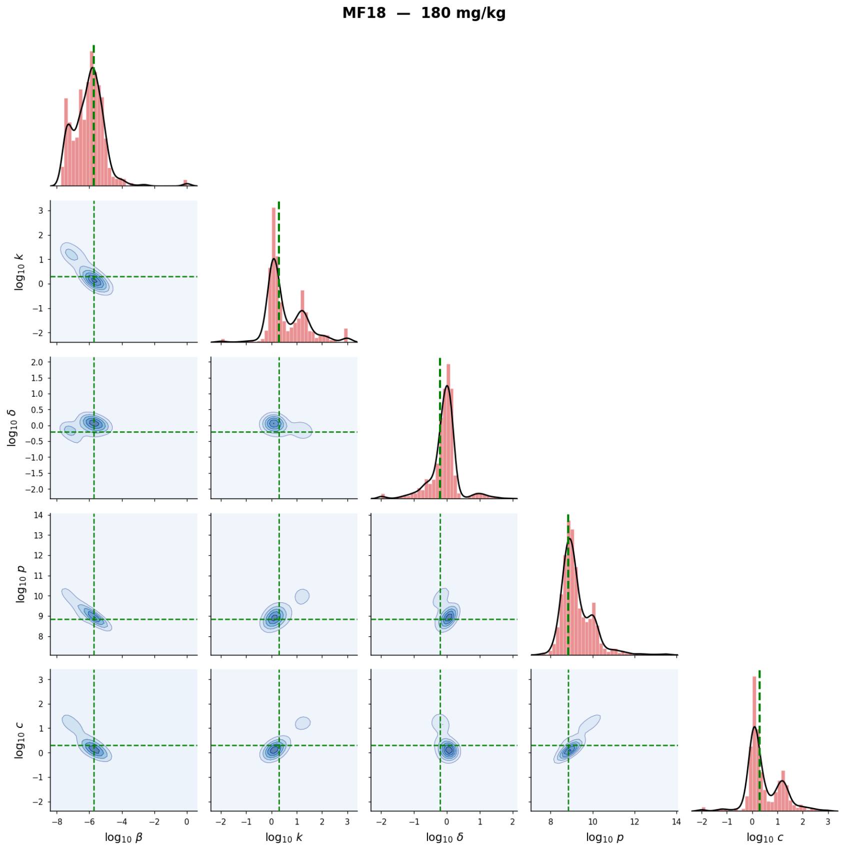

Figure S27: Bootstrap parameter distributions for animal MF18 (180 mg/kg).

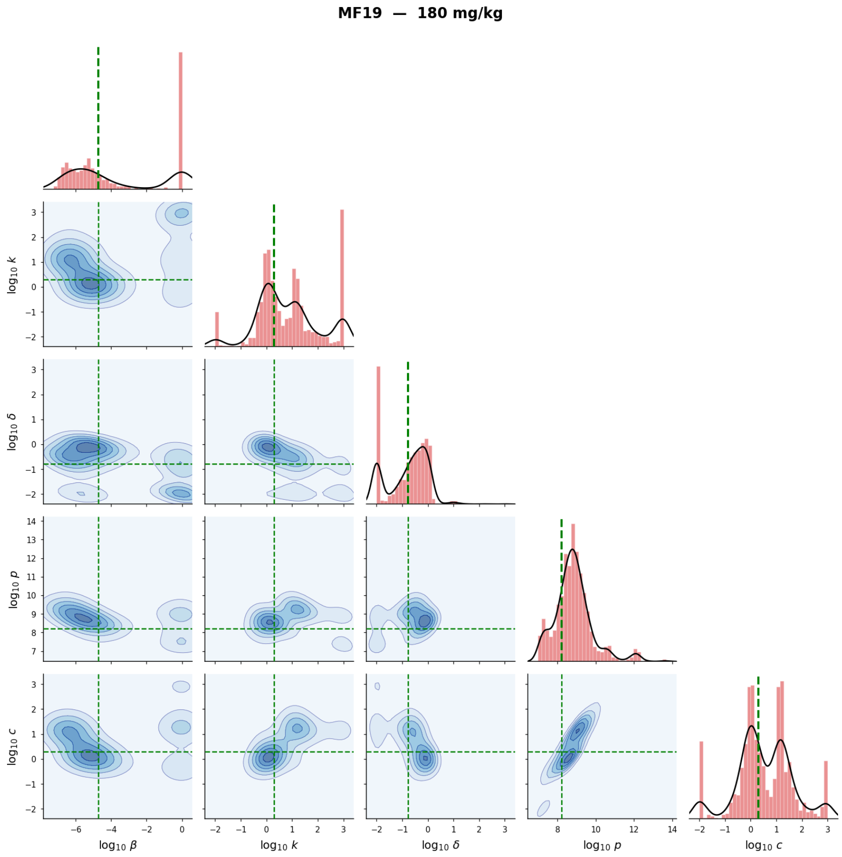

Figure S28: Bootstrap parameter distributions for animal MF19 (180 mg/kg).

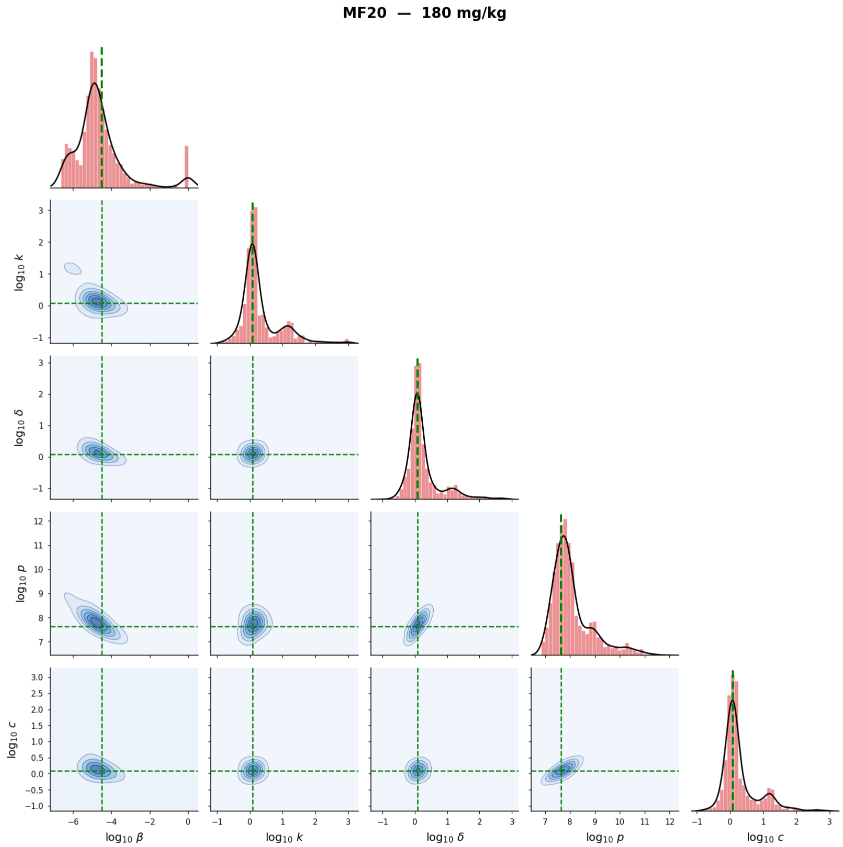

Figure S29: Bootstrap parameter distributions for animal MF20 (180 mg/kg).

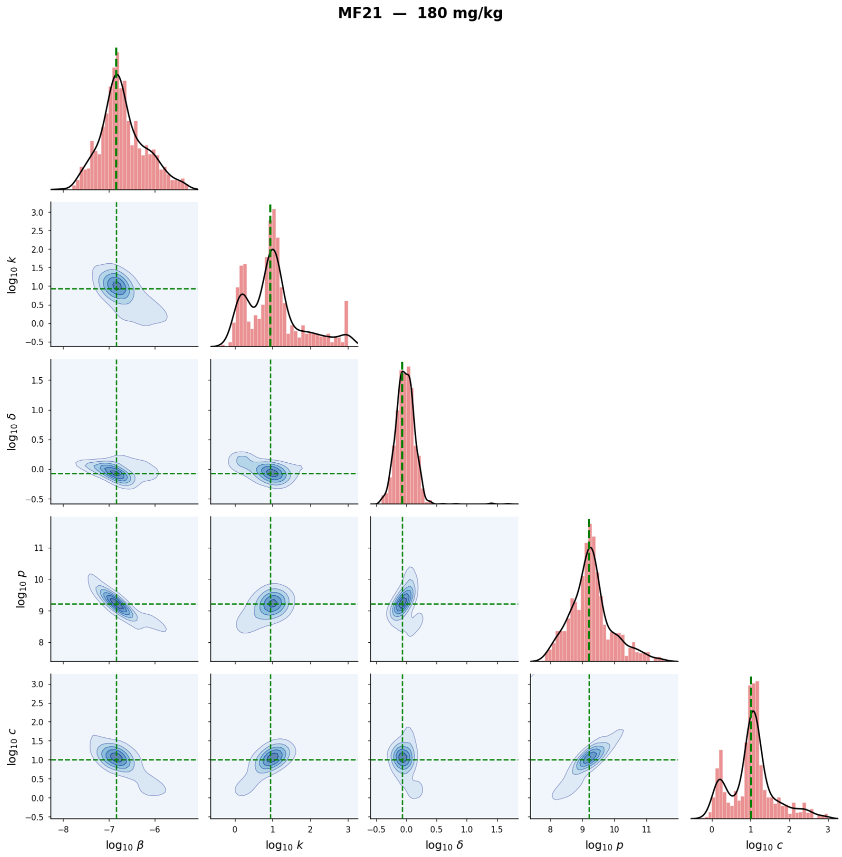

Figure S30: Bootstrap parameter distributions for animal MF21 (180 mg/kg).

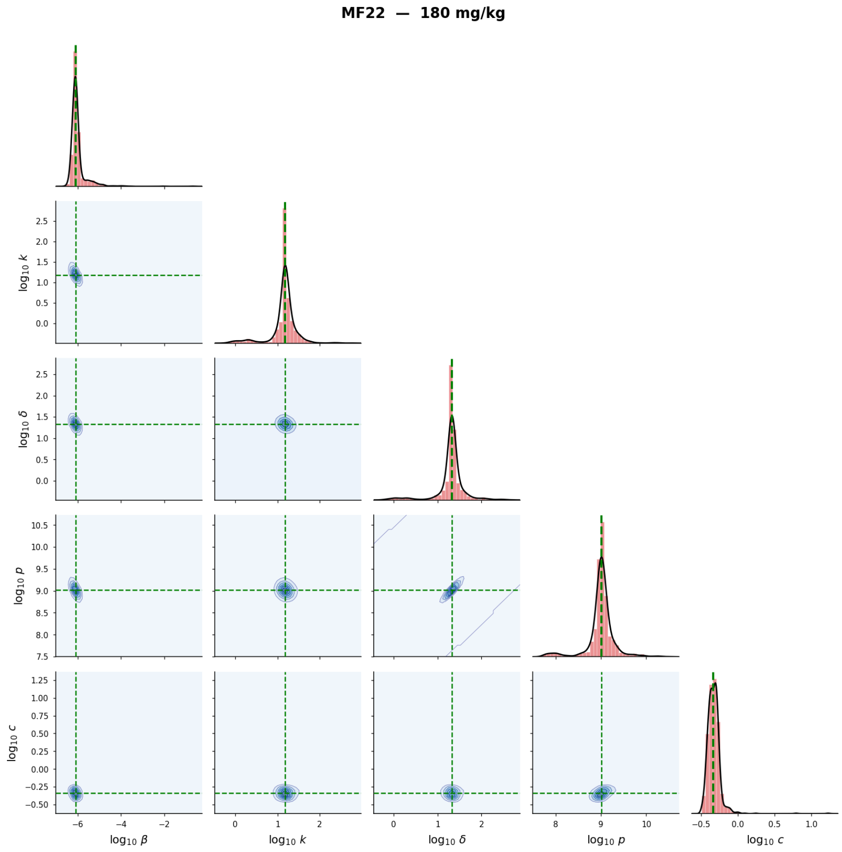

Figure S31: Bootstrap parameter distributions for animal MF22 (180 mg/kg).

S3 Group Corner Plots

The following corner plots show bootstrap parameter distributions for the geometric mean viral load trajectory of each treatment group. The geometric mean trajectory for each group was treated as a single data series, and 1 000 bootstrap replicates were generated by resampling residuals from the best fit, in the same manner as the individual animal analyses in S1. Green dashed lines indicate the maximum-likelihood parameter values.

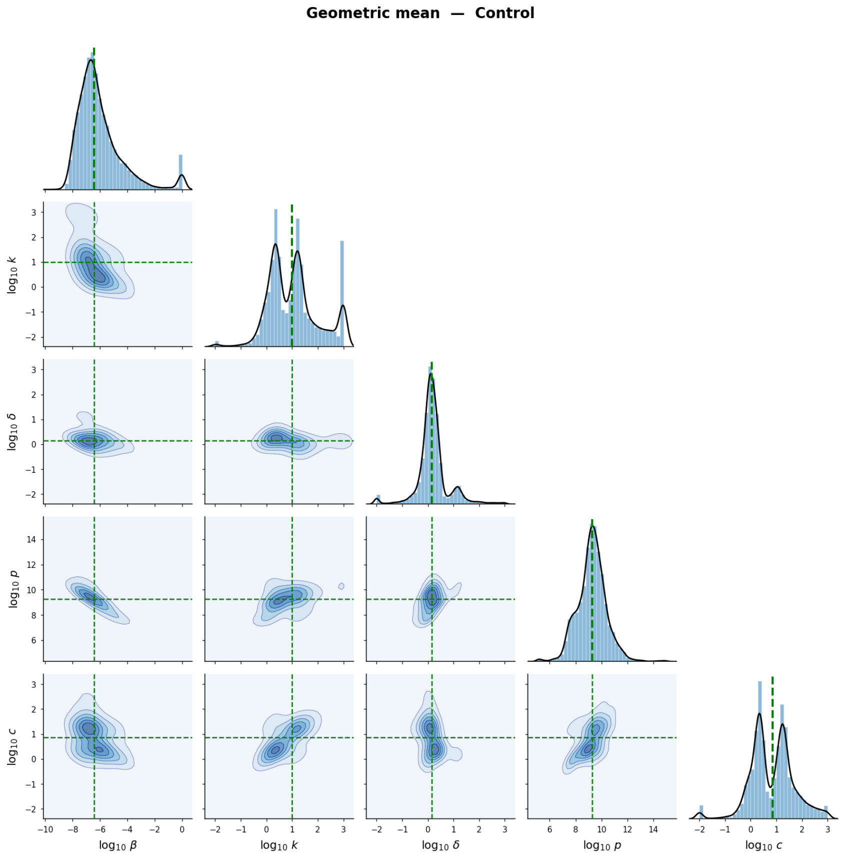

Figure S32: Bootstrap parameter distributions for the Control group geometric mean trajectory (1 000 bootstrap replicates).

Geometric mean — 100 mg/kg

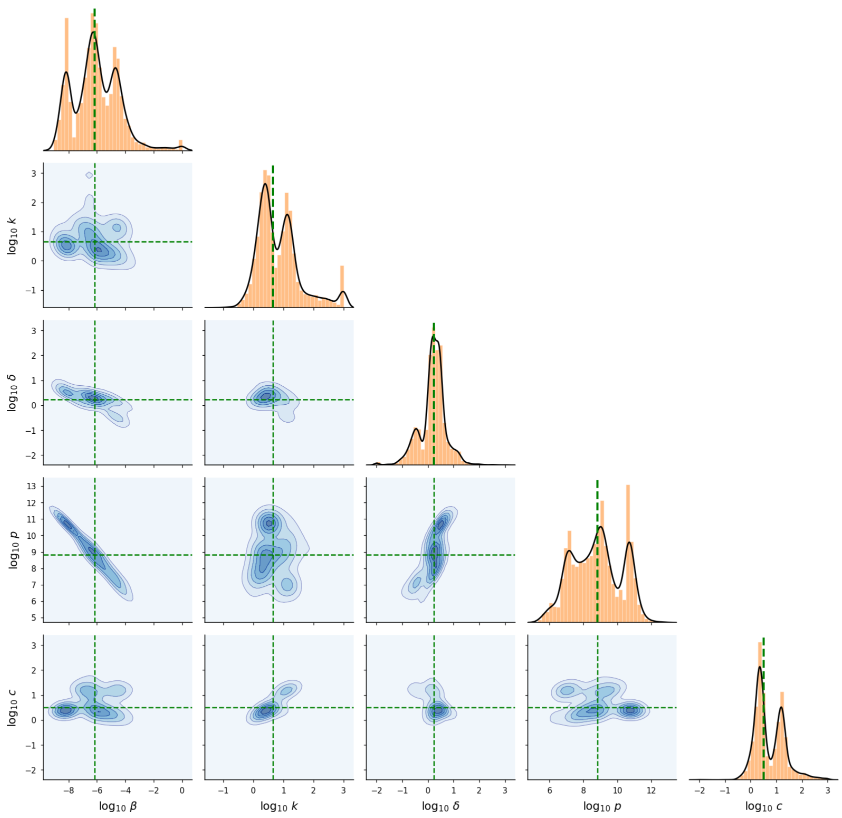

Figure S33: Bootstrap parameter distributions for the favipiravir 100 mg/kg group geometric mean trajectory (1 000 bootstrap replicates).

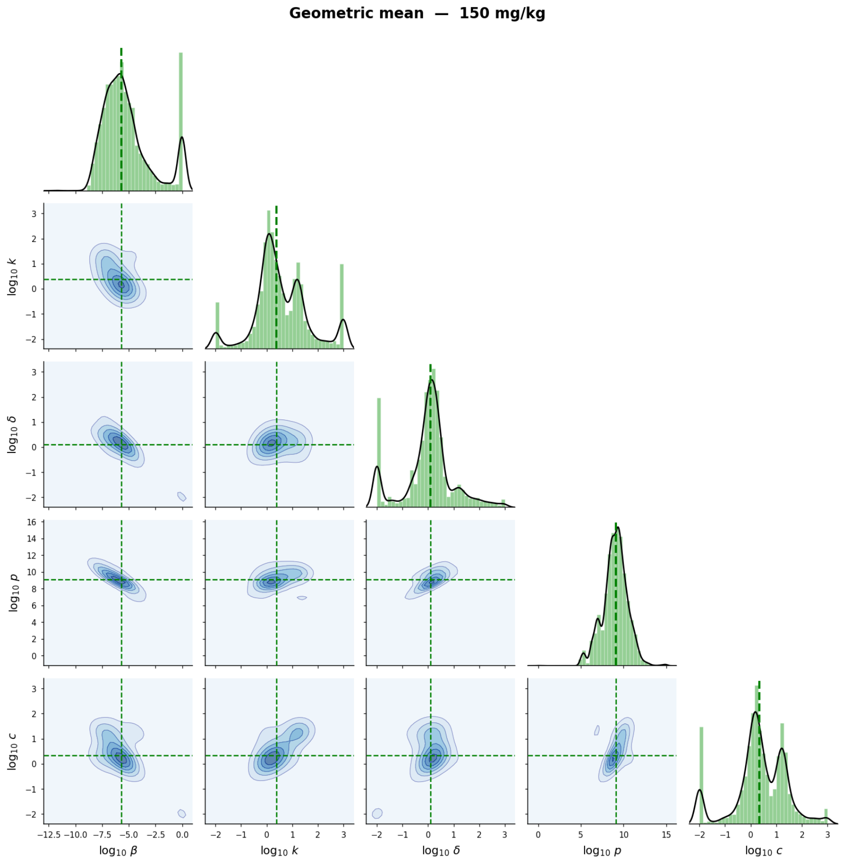

Figure S34: Bootstrap parameter distributions for the favipiravir 150 mg/kg group geometric mean trajectory (1 000 bootstrap replicates).

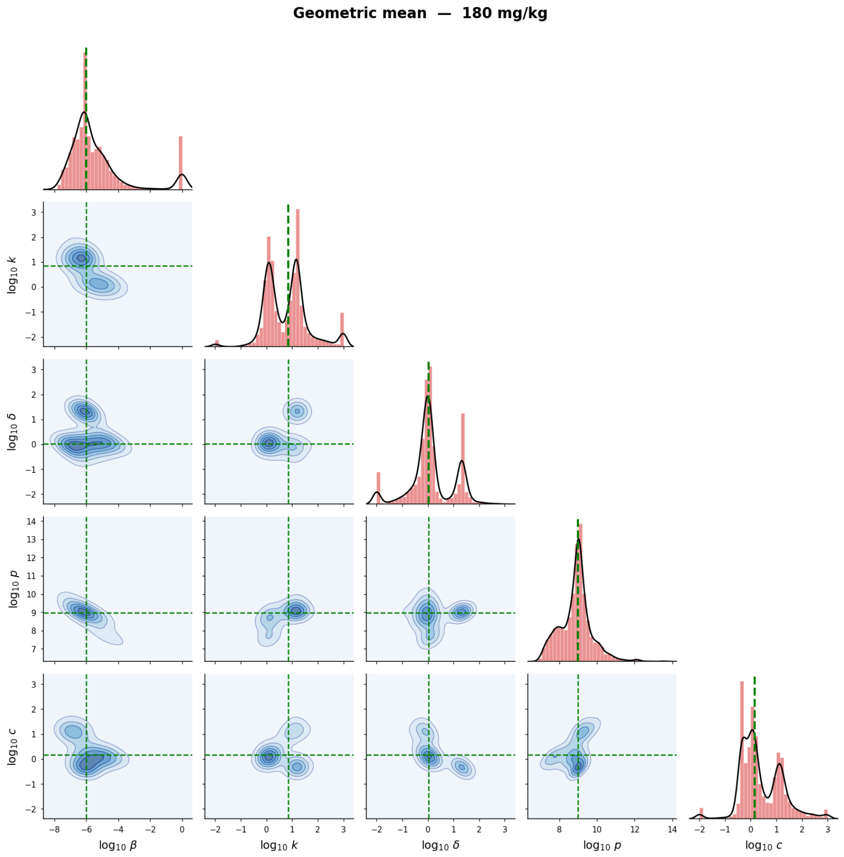

Figure S35: Bootstrap parameter distributions for the favipiravir 180 mg/kg group geometric mean trajectory (1 000 bootstrap replicates).
